# Supplementary material for: Long-term neurological complications in COVID-19 survivors: study protocol of a prospective cohort study (NeurodegCoV-19)
Source: BMJ Open. 2023 Jul 30;13(7):e072981. doi: 10.1136/bmjopen-2023-072981 (PMC10387627; doi:10.1136/bmjopen-2023-072981)
Supplement: Author's manuscript [file bmjopen-2023-072981.draft_revisions.pdf]

BMJ Open is committed to open peer review. As part of this commitment we make the peer review history of every article we publish publicly available.

When an article is published we post the peer reviewers' comments and the authors' responses online. We also post the versions of the paper that were used during peer review. These are the versions that the peer review comments apply to.

The versions of the paper that follow are the versions that were submitted during the peer review process. They are not the versions of record or the final published versions. They should not be cited or distributed as the published version of this manuscript.

BMJ Open is an open access journal and the full, final, typeset and author-corrected version of record of the manuscript is available on our site with no access controls, subscription charges or pay-per-view fees (http://bmjopen.bmj.com).

If you have any questions on BMJ Open's open peer review process please email info.bmjopen@bmj.com

Long-term neurological complications in COVID-19 survivors: study protocol of a prospective cohort study (NeurodegCoV-19).

Journal:	*BMJ Open*
Manuscript ID	bmjopen-2023-072981
Article Type:	Protocol
Date Submitted by the Author:	19-Feb-2023
Complete List of Authors:	Araujo, Natalia; UP EPIUnit; UP ISPUP ITR, Departamento de Ciências da Saúde Pública e Forenses e Educação Médica<br>Silva, Isa; UP EPIUnit; UP ISPUP ITR<br>Campos, Patrícia; UP EPIUnit; UP ISPUP ITR<br>Correia, Rita; UP EPIUnit; UP ISPUP ITR<br>Calejo, Margarida; ULSM<br>Freitas, Pedro; IPP ESS<br>Seco, Mariana; ULSM<br>Ribeiro, Luís; ULSM<br>Costa, Ana ; University of Porto Epidemiology Research Unit; UP ISPUP ITR<br>Morais, Samantha; University of Porto Epidemiology Research Unit; UP ISPUP ITR<br>Pereira, Susana; University of Porto Epidemiology Research Unit; Francisco Gentil Portuguese Institute for Oncology of Porto, Neurology<br>Firmino-Machado, João; UP EPIUnit, EPIUnit; UP ISPUP ITR<br>Rodrigues, Rita; CHEDV<br>Pais, Joana; UP EPIUnit; UP ISPUP ITR<br>Ruano, Luís; UP EPIUnit; CHEDV<br>Lunet, Nuno; FMUP, Department of Clinical Epidemiology, Predictive Medicine and Public Health; UP EPIUnit<br>Tedim-Cruz, Vítor; UP EPIUnit; ULSM
Keywords:	COVID-19, Dementia < NEUROLOGY, Neurological injury < NEUROLOGY, Parkinson-s disease < NEUROLOGY, EPIDEMIOLOGIC STUDIES

SCHOLARONE™
Manuscripts

Title

Long-term neurological complications in COVID-19 survivors: study protocol of a prospective cohort study (*NeurodegCoV-19)*.

Natália Araújo[1,2,3], Isa Silva[1,2], Patrícia Campos[1,2], Rita Correia[1,2], Margarida Calejo[4], Pedro Freitas[5], Mariana Seco[4], Luís Ribeiro[4], Rute Costa[1,2,3], Samantha Morais[1,2], Susana Pereira[1,2,6], Firmino Machado[1,2], Rita Rodrigues[7], Joana Pais[1,2], Luís Ruano[1,2,3,7], Nuno Lunet[1,2,3], Vítor Tedim-Cruz[1,2,4]

[1] EPIUnit – Instituto de Saúde Pública, Universidade do Porto, Rua das Taipas, 135, 4050-600 Porto, Portugal

[2] Laboratório para a Investigação Integrativa e Translacional em Saúde Populacional (ITR), Rua das Taipas, 135, 4050-600 Porto, Portugal

[3] Departamento de Ciências da Saúde Pública e Forenses e Educação Médica, Faculdade de Medicina da Universidade do Porto, Alameda Professor Hernâni Monteiro, 4200-319 Porto, Portugal

[4] Serviço de Neurologia, Unidade Local de Saúde de Matosinhos, 4464-513 Matosinhos, Portugal

[5] Escola Superior de Saúde do Politécnico do Porto, Rua Dr. António Bernardino de Almeida, 400, 4200-072, Porto, Portugal

[6] Instituto Português de Oncologia do Porto, Rua Dr. António Bernardino de Almeida, 4200-072 Porto, Portugal

[7] Serviço de Neurologia, Centro Hospitalar Entre Douro e Vouga, 4520-211 Santa Maria da Feira, Portugal

Corresponding author

Natália Araújo
Rua das Taipas, 135
4050-600 Porto, Portugal
Tel. +351 222 061 820
natalia.araujo@ispup.up.pt

Words count: 4218 (4014 words in the main text: Introduction, Methods and Analysis, Ethics and dissemination)

For peer review only - http://bmjopen.bmj.com/site/about/guidelines.xhtml

Abstract

Background

Evidence suggests an association between SARS-CoV-2 infection and worse performance on cognitive tests, and a higher risk of Parkinson's disease (PD) and dementia up to six and 12 months after infection, respectively. Longer follow-ups with comparison groups are needed to clarify the potentially increased risk of neurodegenerative diseases in COVID-19 survivors, namely those infected before mass vaccination.

Methods

A prospective study started in July 2022 with four cohorts of 150 individuals each, defined according to SARS-CoV-2 infection and hospitalization status between March 2020 and February. 2021: cohort 1—hospitalized due to SARS-CoV-2 infection; cohort 2—hospitalized, COVID-19-free; cohort 3—infected, not hospitalized; cohort 4—not infected, not hospitalized. Cohort 2 will be matched to cohort 1 according to age, sex, level of hospitalization care, and length of stay; cohort 4 will be age- and sex-matched to cohort 3. Baseline, one- and two-year follow-up evaluations will include: cognitive performance assessed with the Montreal Cognitive Assessment (MoCA) and neuropsychological tests; the assessment of prodromal markers of PD with Rapid Eye Movement Sleep Behavior Disorder single-question Screen and self-reported olfactory and gustative alterations; screening of PD with the 9-item PD screening questionnaire; gait evaluation with Timed Up&Go test. Suspected cases of cognitive impairment and PD will undergo a clinical evaluation by a neurologist. Frequency measures of neurological complications, prodromal markers, and diagnoses of dementia and PD, will be presented. The occurrence of cognitive decline— the difference between baseline and one-year MoCA scores below 1.5 standard deviations below the mean of the distribution of the variation— will be compared between cohorts 1 and 2, and cohorts 3 and 4 with Odds Ratio estimated using multivariate logistic regression.

Ethics and dissemination

This study received ethics approval and results will be disseminated among the scientific community and the public.

For peer review only - http://bmjopen.bmj.com/site/about/guidelines.xhtml

Strengths and limitations of this study

- The occurrence of neurological complications will be assessed after an even longer period after SARS-CoV-2 infection than in previous studies, that is after two to three years and afterward, yearly for two years.
- Prodromal markers of Parkinson's disease and dementia, as well as diagnoses of these diseases, will be evaluated with screening tests, neuropsychological tests, and a clinical evaluation by a neurologist.
- In addition to episodic cognitive impairment, we will assess cognitive performance over two years and compare individuals who had mild/severe SARS-CoV-2 infections with the general population or a group of hospitalized patients.
- Sample size, calculated to detect an association between SARS-CoV-2 infection and cognitive decline, may not be large enough to identify a significant association between COVID-19 and dementia or PD, requiring a longer follow-up.
- The comparison groups were COVID-19-free between March 2020 and February 2021 but some may have been infected afterward after vaccination, resulting in a possibly weaker association than if comparison groups had been completely COVID-19 over all the study period.

Keywords

COVID-19; Dementia; Neurological injury; Parkinson's disease; Epidemiologic studies

For peer review only - http://bmjopen.bmj.com/site/about/guidelines.xhtml

Introduction

COVID-19 mortality is still high in some countries and the response to the pandemic is not uniform around the world. Mass vaccination, booster vaccine doses offered to the most vulnerable population subgroups, access to adequate treatment of COVID-19, and less aggressive variants have allowed many countries to face new cases of SARS-CoV-2 infection with a lower load on the health system (1). However, the long-term health consequences of SARS-CoV-2 or long COVID may represent an important health problem. Long COVID is defined by the World Health Organization as the continuation or development of new symptoms three months after the initial SARS-CoV-2 infection, with these symptoms lasting for at least two months with no other explanation (2). It may affect 45% of hospitalized and non-hospitalized SARS-CoV-2 infected individuals within four months post-infection (3), and its worldwide prevalence at 120 days from infection, was estimated at 0.49% (4), accounting for nearly 38 million cases. Fatigue and cognitive complaints are the most frequent symptoms of long COVID (4) and were associated with difficulties in returning to full-time employment (5).

Although COVID-19 is a respiratory disease, neurological manifestations during the acute phase of SARS-CoV-2 infection are common (6). A few cases of parkinsonism developing between five and 32 days after initial symptoms of SARS-CoV-2 infection were described, suggesting parkinsonism as a consequence of mild and severe COVID-19 infection (7-10). In a large retrospective study (January to December 2020), COVID-19 was also associated with an increased risk of neurologic conditions during the six months following infection, namely, parkinsonism (increased risk in 45%) and dementia (increased risk in 71%), when compared to matched controls of individuals infected with other respiratory tract infections (11). Neurological symptoms after infection have been corroborated with brain microstructural changes on imaging exams, namely in the central olfactory system, three months after recovery from COVID-19, with no differences according to COVID-19 severity (12). Indeed, there is evidence from *in vivo* (humans), *in vitro,* and animal studies, that the novel coronavirus has the potential to be neuroinvasive (13).

Early detection of neurodegenerative complications during prodromal phases of disease is essential for implementing disease modifying interventions. Impaired olfaction and rapid eye movement (REM) sleep behavior disorder (RBD) are prodromal markers with the highest sensitivity and specificity, respectively, to predict Parkinson's disease (PD) (14, 15). Non-dementia cognitive impairment and low gait speed can be used to predict dementia (16-18). Moreover, cognitive decline over one year, that is, a deterioration in cognitive performance over this period, may help in identifying individuals who have normal cognitive performance but who

For peer review only - http://bmjopen.bmj.com/site/about/guidelines.xhtml

will develop a long-term declining trajectory of cognitive performance (19). Therefore, this study protocol describes the ongoing prospective cohort study *NeurodegCoV-19* which aims to:

1) Quantify the association between SARS-CoV-2 infection and one-year cognitive decline, comparing individuals infected with SARS-CoV-2 virus (hospitalized patients or cases with clinical follow-up in the community) and a control group (hospitalized patients or the general population);
2) Describe trajectories of cognitive performance over two years;
3) Estimate frequency measures of the occurrence and persistence of long-term neurologic symptoms and diagnoses, and prodromal markers of PD, in COVID-19 survivors.
4) Explore the associations between SARS-CoV-2 infection and prodromal markers of PD.

In this study, the reference period to define SARS-CoV-2 infection/COVID-19 hospitalization was the first year of the pandemic. Infections in this period are more likely to be associated with an increased risk of neurological complications because vaccines were essentially unavailable and because in vitro and animal models have shown the lower neurotropism and neurovirulence of the Delta and Omicron BA.1 variants (20). Recruitment began on July 01, 2022 and is expected to end in July 2023. Therefore, the baseline evaluation is being performed two to three years after SARS-CoV-2 infection, and the two-year follow-up is expected to be complete in July 2025.

For peer review only

For peer review only - http://bmjopen.bmj.com/site/about/guidelines.xhtml

Methods and Analysis

Setting

This prospective cohort study is based on the population of Matosinhos, one of the counties of the Porto Metropolitan Area, Northern Littoral of Portugal, the region with the highest number of COVID-19 cases during the first months of the pandemic (326 378 cases until February 28, 2021) (21). As nearly all Portugal dwellers are enrolled in public primary healthcare units, those enrolled at these units of Matosinhos (176 299 inhabitants enrolled in 2020, and 177 715 in 2021) were the source population for this study. The local health unit of Matosinhos, *Unidade Local de Saúde de Matosinhos* (ULSM), integrates primary and continuous palliative care units, and its hospital, *Hospital Pedro Hispano,* under a single entity and the same management. The clinical pathology laboratory of ULSM performed all COVID-19 diagnostic tests prescribed by family doctors and public health services of the ULSM, except when results could not be obtained in 24 hours due to an overload of testing; private laboratories collaborated in the latter cases.

Additionally, the hospital unit *Centro Hospitalar de Entre Douro e Vouga (CHEDV)* in *Santa Maria da Feira*, another county of the Porto Metropolitan Area, which includes three hospitals—*Hospital de São Sebastião, E.P.E.* (in *Santa Maria da Feira*), *Hospital Distrital de São João da Madeira*, and *Hospital São Miguel* (in *Oliveira de Azeméis*)— also participated for the selection of hospitalized participants. CHEDV does not include primary health care nor continuous palliative care units.

Study design

This is a prospective study with a baseline assessment two to three years after SARS-CoV-2 infection and follow-up evaluations one and two years later. Baseline assessments started on July 1, 2022, and are expected to end on July, 2023. Participants were retrospectively identified from administrative lists of ULSM and CHEDV, and according to the exposure definition, as follows: having a first diagnosis of SARS-CoV-2 infection (detection of RNA virus in nasopharyngeal or oropharyngeal swabs, with Real-Time Polymerase Chain Reaction), before any vaccination, during the period from March, 2020 (first case diagnosed in Portugal on March 2) to February 2021 [complete vaccination uptake reached 3% and partial vaccination, 6%, on February 28, 2021 (22)]. Exposed individuals are grouped according to hospitalization at *Hospital Pedro Hispano* or at one of the three hospitals of CHEDV due to infection with COVID-19 — cohort 1, or clinical follow-up in the community — cohort 3. The unexposed group is defined as

For peer review only - http://bmjopen.bmj.com/site/about/guidelines.xhtml

having no diagnosis of SARS-CoV-2 infection in the same period, nor after if unvaccinated, and with at least the primary scheme of vaccination, to avoid the inclusion of controls who could get infected during the follow-up period without being vaccinated. Unexposed individuals were also grouped according to having been hospitalized at *Hospital Pedro Hispano* or at CHEDV during the same period— cohort 2, or not — cohort 4.

According to instructions from the Ministry of Health, from October 27, 2020, all patients had to be tested for SARS-CoV-2 infection before hospital admission (23). Therefore, efforts were initially made to select participants infected and/or hospitalized after this date to ensure the non-infection status of the hospitalized comparison group. However, due to a low number of eligible participants for cohort 1, we extended the period to March 2020.

<ins>Participants</ins>

Eligible participants are 18 or older at the COVID-19 diagnosis (similar age at baseline for the comparison groups). General and cohort-specific eligibility criteria are checked by consultation of medical records and complemented with information from the participant during recruitment.

<ins>SARS-CoV-2 infected patients</ins>

Participants with a positive test for SARS-CoV-2 infection between March 2020 and February 2021, were retrospectively identified from the ULSM and CHEDV databases, which, in the case of ULSM, includes tests sampled at the ULSM (96.7%), at temporary emergency units outside the hospital (0.3%,) and the drive-in community center (3.1%), whereas all tests from CHEDV were sampled at the hospitals.

Individuals infected with SARS-CoV-2 were included into one of two groups: cohort 1, individuals hospitalized at *Hospital Pedro Hispano* or at CHEDV due to COVID-19 infection, and cohort 3, individuals with clinical follow-up of COVID-19 in the community (asymptomatic or not). Individuals for cohort 1 were identified in two steps: 1) exclusion, from the list of hospitalization episodes at *Hospital Pedro Hispano* or at CHEDV, of those with a description of Homogeneous Diagnostic Groups surely not compatible with virus infection (e.g., childbirth); 2) consultation of medical records of hospitalization episodes not excluded in the previous step, to identify if hospital admission was due to COVID-19, the highest level of care (general ward, intermediate care, intensive care unit), and length of stay (Figure 1). All individuals identified were contacted for recruitment.

For peer review only - http://bmjopen.bmj.com/site/about/guidelines.xhtml

For peer review only

Individuals for cohort 3 were those on the ULSM list of positive tests for SARS-CoV-2 infection and not on the ULSM list of hospitalized patients (criterion confirmed by the participant) (Figure 1). Three pools of individuals were selected from the pandemic's first, second, and third waves (earliest 30 diagnosis dates in 2020; all cases from October 27 to November 03, 2020, and the latest 30 diagnosis dates in February 2021).

Unexposed comparison groups

A comparison group of hospitalized patients (cohort 2), not infected with SARS-CoV-2 between March 2020 and February 2021, never infected afterwards or infected after complete primary vaccination, was identified from the list of hospitalized patients between March 2020 and February 2021 who were not on the list of positive tests for COVID-19 for this period (Figure 1). Individuals for cohort 2 will be matched with individuals from cohort 1 regarding the hospital (*Hospital Pedro Hispano or* CHEDV), age (18-24, 25-34, 35-44, 45-54, 55-64, 65-74, and ≥75 years), sex, level of hospitalization care (general ward, intermediate care, intensive care unit), number of days of hospitalization (1, 2-7, 8-15, 16-30, and >30 days), and month of hospital admission.

For comparison with cohort 3, cohort 4 will include (baseline) age- and sex-matched individuals randomly selected from the pool of individuals enrolled at ULSM, who were not on the list of a positive diagnostic test for COVID-19 between March 2020 and February 2021, nor on the list of hospitalized patients in the same period (Figure 1).

Exclusion criteria

We will exclude: 1) individuals diagnosed with a condition impairing cognitive function or enrolled in treatments for cognitive decline (consumption of Memantine, Rivastigmine, or Donepezil; participation in psychotherapeutic interventions for clinical or research purposes); 2) individuals with less than one year of formal education, and primary education (grades one to four) not in the Portuguese education system (criteria for the use of cognitive tests); 3) individuals with a motor, visual or auditory problem incompatible with the performance of the cognitive tests.

Individuals living at senior homes are excluded, as well as those infected between the two doses of the primary vaccination scheme or within the 14 days following vaccination.

For peer review only - http://bmjopen.bmj.com/site/about/guidelines.xhtml

Recruitment

Phone calls to contact the participants are performed during the day (morning, afternoon and evening) and with repeated attempts, in case of failure. A trained member of the research team conducts a structured conversation aiming to confirm the eligibility of participants, explain the objectives and procedures of the study, and schedule the in-person evaluation at *Hospital Pedro Hispano* or *Hospital São Sebastião*. Reasons for not participating (impossible to contact, exclusion criteria or refusals) are registered for all potential participants identified for the cohorts 1 to 4.

Baseline and follow-up evaluations

Participants are evaluated in person, at *Hospital Pedro Hispano* or *Hospital São Sebastião*, at baseline, that is, approximately two to three years after the index date, which corresponds to the date of the first positive test for SARS-CoV-2 infection, for cohort 3, the date of hospital admission due to acute COVID-19, for cohort 1, and the date of hospital admission due to other reasons, for cohort 2. No index date was attributed to participants of cohort 4 as they were neither infected nor hospitalized during the period March, 2020 to February, 2021.
Follow-up evaluations after one and two years will be similar to the baseline assessment.

Instruments

Table 1 presents the instruments administered to all participants of the four cohorts.

Structured questionnaire

Trained interviewers conduct face-to-face interviews using a structured questionnaire to collect baseline data on: 1) sociodemographic characteristics: age, years of education, employment status, occupation; 2) lifestyles: alcohol and tobacco consumption; 3) previous medical diagnoses and current medication intake; 4) neurologic symptoms during infection and following recovery (cohorts 1 and 3), and current symptoms (all participants); 5) dates of SARS-CoV-2 infection diagnoses (re-infections for cohorts 1 and 3, and infection episodes after complete vaccination, for cohorts 2 and 4); 6) data on vaccination uptake.

Evaluation of cognitive performance and identification of cognitive impairment — Montreal Cognitive Assessment and Brain on Track, neuropsychological evaluation, and clinical evaluation by a neurologist

For peer review only - http://bmjopen.bmj.com/site/about/guidelines.xhtml

Cognitive performance of all participants is evaluated with the Montreal Cognitive Assessment (MoCA), version 7.1 (24), translated, adapted and validated in the Portuguese population (25). MoCA is a screening instrument for mild cognitive impairment, with good psychometric characteristics, and high sensitivity (26). It assesses visuoconstructive ability, language, attention, concentration and working memory, executive functions, delayed memory, and orientation. Scores range 0 to 30, with higher values corresponding to better cognitive performance. Interviewers received training and certification from the MoCA Training and Certification Module at www.mocacognition.com, and the Manual of the Portuguese version 7.1 was discussed within the research team to clarify administration procedures and the rating process.

Two outcome measures will be used: cognitive decline and probable cognitive impairment. We will define the former based on the distribution of the MoCA scores variation over time (score at one-year *minus* score at baseline) in the comparator non-exposed group (either cohort 2 or cohort 4), as being a variation in MoCA scores below 1.5 standard deviations (SD) below the mean. For defining probable cognitive impairment, we use age- and education-specific norms (26) and the cut-off point of 1.5 SD below the mean.

Participants with probable cognitive impairment are invited for a neuropsychological assessment, to be scheduled as soon as possible, to confirm cognitive deficits. The neuropsychological evaluation includes neurocognitive tests validated for the Portuguese population, assessing verbal and visual memory, working memory, information processing speed, executive functions, and language. It also includes the assessment of subjective memory complaints, emotional functioning, functional disability, independent living skills, and pre-morbid intelligence (Table 2).

Based on the number of tests used for evaluating each cognitive domain, cut-off points (below 1 SD, 1.5 SD or 2 SD below the mean of age-norms) are applied to identify cognitive deficit (27) (Table 3). Participant with cognitive deficit in at least one cognitive domain are considered to have cognitive impairment. These are referred to a clinical consultation with a neurologist of the research team at the hospital, for clinical follow-up according to the best clinical practices in use and a structured protocol of classification of the probable etiology of the cognitive impairment.

Additionally, cognitive performance is evaluated with the web-based remote monitoring test Brain on Track. This instrument showed good internal consistency, discriminative ability and reliability (28, 29). It assesses executive functions, short and delayed memories, attention, calculation, visuoconstructive ability, and language. A complete session of tests of Brain on Track takes 24 minutes and includes 11 exercises with fixed duration. The participant is asked to answer to as many items as possible in each exercise, as the score is the number of correct

For peer review only - http://bmjopen.bmj.com/site/about/guidelines.xhtml

answers. Based on this score and using a predictive model adjusted for age (to be published), a score of risk for cognitive impairment is computed. Participants with a high score of risk will be invited for the neuropsychological assessment.

Eligibility criteria to use Brain on Track are: at least three years of education, access to a computer at home with internet, and sufficient digital literacy to log into the website, or with help from another person but with autonomy for using the mouse and to do the exercises independently.

All participants eligible to use Brain on Track are invited for remote monitoring. The first and second sessions of tests are scheduled with a one-week interval, and the following sessions every three months thereafter.

For peer review only

Identification of prodromal markers and screening for Parkinson's disease

REM Sleep Behavior Disorder Single-Question Screen

We use the REM Sleep Behavior Disorder Single-Question Screen (30) to identify idiopathic rapid eye movement sleep behavior disorder (RBD), which is an important risk factor for PD (31). RBD may be considered a prodromal marker of PD, dementia with Lewy Bodies, and multiple system atrophy, and about 75% of those suffering from RBD develop PD or a parkinsonism within about 10 years (32). This rapid test has high sensitivity, specificity and reliability (30).

The nine-item Parkinson's disease screening questionnaire

We use the nine-item PD screening questionnaire (33) which has a sensitivity and a specificity ranging 61-92% and 29-92%, respectively, to identify possible cases of PD in community settings (34). The test includes nine questions to be answered with *Yes* or *No.* The sum of positive answers is the final score of the test which defines the likelihood of having PD: 0–1 (unlikely), 2–4 (possible), ≥ 5 (probable) (34).

Participants screening positive for possible parkinsonism are formally evaluated by a neurologist of the research team, following usual clinical practices.

Timed "Up and Go" test and gait assessment

We use the Timed "Up and Go" Test (35) to measure the ability of participants to perform sequential locomotor tasks that incorporate walking and turning. This test was designed to measure mobility in elderly people and could be a useful tool for quantifying locomotor performance in people with PD. Patients are required to stand up from a chair, walk three

For peer review only - http://bmjopen.bmj.com/site/about/guidelines.xhtml

meters, turn around, walk back to the chair, and sit down again. One training trial is performed under instructions of a trained member of the research team and then two trials are videotaped to allow a trained neurologist of the research team to later evaluate gait and balance. The time, in seconds, necessary for the participant to perform the test is recorded. The two videotaped trials of the TUG test are evaluated using a structured report comprising: 1) four gait-related features (ability to stand up from a chair, walking characteristics, presence of "freezing" walking, posture); 2) the rating of arms swing, asymmetry of shoulder lift, and tremor at rest on gait; 3) and a global gait diagnosis ("normal" or "altered", with parkinsonism and/or osteoarticular and/or vascular and/or others features).

Assessment of anxiety, depression, sleep quality, and obstructive sleep apnea

Levels of anxiety and depression symptoms are measured using the Hospital Anxiety and Depression Scale (HADS) (36) validated in the Portuguese population (37). This self-administered questionnaire includes seven items assessing anxiety symptoms and seven items for depression symptoms, resulting in the HADS-A and HADS-D subscales, respectively, rated with a four-point Likert scale (0 to 3). Scores of 11 or higher in the subscale are indicative of clinically significant symptoms (37). HADS was designed to be used in patients with physical illness, and for this reason, the questionnaire does not include somatic symptoms, namely insomnia, loss of appetite and fatigue, to avoid overlapping of symptoms due to depression and due to physical conditions. Several studies have shown its suitability in both clinical and community settings (38).

To assess sleep quality, we use the self-administered questionnaire Pittsburgh Sleep Quality Index (39), validated in the Portuguese population (40). This instrument presents 19 items related to seven components — subjective sleep quality; sleep latency; sleep duration; habitual sleep efficiency; sleep disturbances; use of sleeping medication; day-time dysfunction. Patients are classified as poor or good sleepers (score equal or higher *vs.* lower than five, respectively).

We use the STOP-Bang questionnaire (41) translated and validated in the Portuguese population (42, 43) to screen obstructive sleep apnea. This test includes eight questions of *Yes* or *No* answer. Three or more positive answers are indicative of obstructive sleep apnea with a sensitivity of 98.6%, and eight positive answers suggest a severe condition, with a sensitivity of 80%, according to estimates obtained in a primary care setting (43). The STOP-Bang questionnaire will also be used to control for the presence of obstructive sleep apnea as mimic of RBD.

For peer review only

For peer review only - http://bmjopen.bmj.com/site/about/guidelines.xhtml

Data analyses and sample size

Outcome measures

We will report on the following outcomes: neurologic symptoms; prodromal markers of PD; probable cognitive impairment detected with the MoCA; cognitive decline detected with the MoCA; impairment in cognitive domains and overall cognitive impairment identified with the neuropsychological assessment; cognitive impairment and PD diagnoses by neurologist.

Frequency measures of the above-mentioned outcomes will be estimated: prevalence at baseline, and after one and two years; period prevalence over one and two years, and cumulative incidence, which will be estimated at one and two years, considering, death as a competitive event, according to the Kalbfleisch and Prentice method (44).

Associations between SARS-CoV-2 infection and cognitive outcomes will be estimated using Odds Ratios (OR) or Hazard Ratios (HR) with their corresponding 95% confidence interval (95%CI), obtained from multivariate logistic and Cox regressions, respectively, and considering sex, age, education, co-morbidities, and disease severity (not hospitalized, hospitalized in ICU, intermediate or general ward, and duration of hospitalization) as co-variables.

The contribution of anxiety and depression symptoms, sleep quality, and obstructive sleep apnea to cognitive decline will also be evaluated using multivariate logistic regression.

Mixed-effect models will be adjusted to compare cognitive trajectories (considering age and education) over two years, according to sociodemographic and clinical characteristics, for each of the cohorts.

Sample size calculation

In order to compare two cohorts of similar disease severity (among hospitalized – cohorts 1 and 2, or among the community – cohorts 3 and 4), considering the occurrence of cognitive decline of 7 per 100 person-years in the non-COVID-19 participants (variation in MoCA scores below 1.5 standard deviation of the normal distribution of the variation), a ratio exposed:unexposed of 1:1, the statistical power of 80% and 5% significance level, 600 participants will be needed (150 in each cohort) to estimate the associations corresponding to a OR of at least 2.5.

This sample size will also be adequate to estimate the frequency of outcomes equal to or lower than 10% with 95% CI, and a margin of error of 7%, as well as incidence rates as low as up to 3.5 per 100 person-years with 95%CI up to 3 per 100 person-years wide.

Patient and Public Involvement Statement

For peer review only - http://bmjopen.bmj.com/site/about/guidelines.xhtml

For peer review only

Participants were not involved in the planning of the research.

For peer review only

For peer review only - http://bmjopen.bmj.com/site/about/guidelines.xhtml

Ethics and dissemination

This study received approval from the Ethics Committee of ULSM (117/CES/JAS), and CHEDV (39/2022), and from the ULSM Local Unit of Data Protection and Security (030/CLPSI/2021), as well as from the Data Protection Officer of the *Instituto de Saúde Pública da Universidade do Porto*. A Data Protection Assessment Impact was performed to evaluate and understand the processing, the need and proportionality of data processing. Therefore, this study complies with the World Medical Association Declaration of Helsinki and the General Data Protection Regulation.

Participants are informed about the institutions involved in the project and its research members; the objectives and procedures of the research; data to be collected, namely clinical data from their electronic health record, questionnaires, cognitive tests, and a video; expected individual benefits and risks (minimal); procedures to respect their right to confidentiality, their right and how to manifest this right, to abandon the study and withdraw their consent at any time and that this will not interfere with their medical care, and their right to access their data and have their data corrected and erased. After this explanation, patients clearly manifesting that they do not understand the information will not be considered eligible. Participants will have time to decide. Participation will be assumed after a participant signs the informed consent. No direct or indirect incentives for participation will be given. This is an observational study and no risk is expected. Participants with neurologic complications of COVID-19 will be followed as are non-participants of the study, according to clinical practice implemented at the health institution. Therefore, participants will not be submitted to additional risk.

The findings of this project will be submitted for publication in international peer-reviewed journals, and proposed for presentation in relevant national and international conferences, which will allow for the dissemination of the main findings across the medical community. Press releases through mass media will also be issued to promote the dissemination of information relevant to the general population and policy-makers. Furthermore, the project will contribute to the training of researchers through the production of masters' theses and doctoral dissertations.

Author statement

NA, MC, ARC (Rute Costa), SM, SP, FM, JP, LR, NL, VTC contributed for the conception and design of the work, as well as for obtaining funding. NA, ARC, IS and PC contributed for the design of the structured questionnaire and interview. IS and PC have recruited the participants. NA, IS, PC, and RC (Rita Correia), have performed the general evaluation of participants. PC and RC have

For peer review only - http://bmjopen.bmj.com/site/about/guidelines.xhtml

performed the neuropsychological evaluation. PF has inserted the data in the database. MS has collected clinical data of the participants. MC, RR, LR, VTC has performed the clinical evaluation by neurologist. NA, IS, PC, RC, MC, JP, LR, NL, VTC will analyze and interpret the data. NA, IS, and PC contributed for drafting the work and all authors revised it critically for important intellectual content. All authors approved the final version of the manuscript to be published.

Data statement

The datasets used and/or analyzed during the current study are available from the corresponding author on reasonable request.

Funding

This study is financed by national funds through the FCT - Foundation for Science and Technology, I.P., within the scope of the projects UIDB/04750/2020 and LA/P/0064/2020, and obtained specific FCT funds through the competitive call for Research and Development projects in all scientific domains (PTDC/SAU-EPI/6275/2020).

Competing interests statement

The authors declare that they have no competing interests.

For peer review only

Table 1. Description of the instruments used for the evaluation of all participants.

Instrument	Description	Domains/subscales	Score
**MoCA (24)**	Paper and pencil cognitive screening test for evaluating mild cognitive impairment.	Attention and concentration; executive functions; memory; language; visuoconstructional skills; calculations; orientation.	Range: 0 to 30<br><br>Higher scores represent better cognitive performance.
**Brain on Track (28, 29)**	Computerized and self-administered test that allows longitudinal monitoring of cognitive performance	Attention; memory; executive functions; language; calculation; constructive ability; visuospatial processing.	Range: virtually unlimited (maximum number of correct answers in a fixed time)<br><br>Higher scores represent better cognitive performance.<br><br>Scores falling below an expected performance threshold for each age/education group represent a pattern of decline in individual performance.
**HADS (40)**	Scale with 14 questions designed to assess anxiety and depression. Patients should answer considering the previous week.	Depression; anxiety.	Range (for each subscale): 0 to 21<br><br>Scores greater than or equal to 11 represent a case of anxiety or depression, as applicable.
**PSQI (39)**	Quality of sleep index with 18 questions about the sleeping habits of the patients during the previous month.	Subjective sleep quality; sleep latency; duration of sleep; habitual sleep efficiency; sleep disorders; use of medications for sleep; daytime dysfunction.	Range: 0 to 21<br><br>Scores greater than five indicate poor sleep quality.

For peer review only

For peer review only - http://bmjopen.bmj.com/site/about/guidelines.xhtml

**REM Sleep Behavior Disorder Single-Question Screen (30)**	Single screening yes/no question about the classic dream-enactment behavior in the REM Behaviour Disorder.	REM sleep behavior disorder.	A positive answer suggests a presence of REM Behaviour Disorder
**STOP-Bang questionnaire (41)**	Screening tool with eight yes or no questions related to the clinical features of sleep apnea.	Obstructive sleep apnea (OSA).	Range: 0 to 8<br>Each positive answer sum 1 to the final score, with higher scores representing more risk of OSA.<br>Scores equal or greater than three indicate
**9-item PD screening questionnaire (33)**	Screening tool with nine yes or no questions	PD	Range: 0 to 9<br>Higher scores indicate higher likelihood of Parkinson' Disease: unlikely if scores are 0–1 ; possible if scores are 2–4; and probable if scores are ≥ 5
**Timed "Up and Go" test (35)**	Test to evaluate the ability of patients to perform sequential locomotor tasks that incorporate walking and turning.	Mobility; gait and balance (ability to stand up from the chair; walking characteristics; presence of "freezing" walking; posture)	5-point Likert scale (normal, discreet, mild, moderate, and severe) and global gait diagnosis: normal or altered, with parkinsonism and/or osteoarticular and/or vascular and/or others features

MoCA – Montreal Cognitive Assessment; BoT – Brain on Track; HADS – Hospital Anxiety and Depression Scale; PSQI – Pittsburgh Sleep Quality Index.

For peer review only

Table 2. Description of the battery of instruments used for the neuropsychological evaluation of the participants with probable cognitive impairment*.

Instrument	Description	Cognitive domains/function	Score
**SMC (45, 46)**	A 10-item scale regarding subjective memory complaints.	Subjective memory.	Range: 0 to 21<br><br>Higher scores reflect maximal memory complaints.
**Verbal Fluency Test (47, 48)**	Verbal fluency tests consist of four trials, one of semantic fluency and three of phonemic fluency, of one minute each. The participants are asked to generate the name of as many species of animals as possible in the semantic fluency test, and as many words as possible beginning with a specific letter in the phonemic fluency test, within one minute.	Non-motor processing speed, language production, and executive functions	The total trial score corresponds to the number of words correctly produced within one minute. The total test score corresponds to the sum of the three trials.<br><br>Higher scores correspond to better performance.
**18-point CDT (49, 50)**	The Clock-Drawing Test is an assessment tool where participants are asked to draw a big circle, place the clock numbers, and then indicate the time as "10 past 11". An 18-point system will be used to score the test.	Visuospatial; executive function.	Range: 0 to 18<br><br>Scoring system with three main components: (a) assessment of circle integrity (two points); (b) number placement and sequencing (six points); and (c) placement and size of the hands (six points). Additionally, there are two points for representation of the clock's center and two points for general gestalt.
**TMT (51, 52)**	Part A: the participants are asked to draw lines to connect 25 randomly scattered numbered circles in ascending order. They should do so as quickly as possible.	Part A: attention; visual scanning; and speed of eye-hand coordination and information processing.	Direct measures of performance: time (seconds) to complete part A and part B and performance errors during part A and part B.

For peer review only

	Part B: the participants are asked to draw lines to connect circles in numeric and alphabetic order, alternating between numbers and letters, progressively up to number 13. They should do so as quickly as possible.	Part B: working memory and executive functions; particularly, the ability to switch between sets of stimuli.	Derived scores: difference score (B - A), ratio score (B/A), proportion score (B - A/A), sum score (A + B), and multiplication score (A × B/100).<br><br>Lower raw scores and higher adjusted scores correspond to better performance.
**WMS-III (53, 54)**	Scale composed of 17 subtests that assess different memory functions using verbal and visual stimuli. Useful in the evaluation of various clinical conditions, including neurodegenerative diseases such as Alzheimer's disease.<br><br>Subtests used: Logical Memory I, II; Visual Reproduction I, II; Digit Span	Verbal and visual memories; working memory.	Range:<br>- Immediate recall: 0-50<br>- Delayed recall: 0-50<br>- Auditory recognition: 0-30<br>- Visual reproduction: 0-104<br>- Digit span: 0-30<br>Higher scores correspond to better performance.
**WAIS-III (55, 56)**	Scale composed of 14 subtests that assess the verbal component and the perceptual-motor component of intelligence in adults and older adolescents.<br><br>Subtests used: Digit-Symbol-Coding; Symbol Search; Information.	Attention/concentration; executive function (sequencing); motor function; processing speed.	The number of correct symbols within the allowed time (120 seconds) is measured.
**Stroop Test (57, 58)**	The Stroop Test evaluates the interference effects between the two cerebral hemispheres. Consist in three trials: 1) word reading; 2) colours naming and 3) identifying the colours of words, without considering the meaning of the word.	Executive functions (inhibitory control); selective attention.	Scores for each trial indicate the number of correct responses. An interference score can be generated that quantifies the participant's ability to inhibit the inappropriate response of reading the colour name as opposed to the colour of the ink used to print the colour name in the third trial.
**Token Test – short form (59)**	A test designed to assess the comprehension of commands that vary in degree of linguistic	Attention and vigilance; verbal functions.	Range: 0-36

For peer review only - http://bmjopen.bmj.com/site/about/guidelines.xhtml

	difficulty. The participant is presented with tokens of different geometric shapes, sizes, and colours, and is required to perform certain acts with the tokens, such as point to tokens, touch them, pick them up, and place one token on top of another.		
**TeLPI (60)**	Designed to assess premorbid intelligence, TeLPI is a Portuguese irregular word reading test. The participants are asked to read 46 irregular words, infrequent Portuguese words.	Premorbid Intelligence Quotient (IQ): full scale IQ; Verbal IQ; performance IQ	Range: number of errors (maximum of 46) and years of education are inserted in three linear equations to calculate the three types of IQ
**BDI-II (61, 62)**	A 21-question measure assessing the presence of depressive symptoms experienced by the participant within the past week.	Emotional functioning.	Range: 0 to 63<br><br>A cut-off score indicative of mild depressive symptoms is greater than 10 and for severe depressive symptoms is greater than 30.
**Barthel ADL Index (63, 64)**	An index to measure functional disability, focused on bodily oriented personal care.	Functional domains: feeding; incontinence; transferring; toileting; dressing; bathing.	Range: 0 to 100<br><br>Lower scores reflect increased disability.
**IADL (65, 66)**	An eight-item scale used to assess independent living skills which include more complex activities (i.e. "instrumental activities of daily living") necessary for functioning in community settings.	Functional domains: using the telephone; shopping; food preparation; housekeeping; laundry; transport; medication; finances.	Range: 0 to 8<br><br>Higher scores reflect high function, independence.

Barthel ADL Index – Barthel Activities of Daily Living Index; BDI-II – Beck Depression Inventory – Second Edition; CDT – Clock Drawing Test; IADL – Lawton and Brody Instrumental Activities of Daily Living; SDMT – Symbol and Digit Modalities Test; SMC – Subjective Memory Complains scale; TeLPI – Irregular Word Reading Test; TMT – Trail Making Test; WAIS-III – Wechsler Adult Intelligence Scale – Third Edition; WMS-III – Wechsler Memory Scale – Third Edition.

[*] Participants with a MoCA score below age- and education-specific values based on norms and the cut-off point of 1.5 standard deviations below the mean

For peer review only

For peer review only - http://bmjopen.bmj.com/site/about/guidelines.xhtml

Table 3. Criteria used for the classification of cognitive impairment, considering the number of tests administered to assess each cognitive domain.

Cognitive domain	Test	Criteria for impairment
**Verbal memory**	WMS III – Logical memory I and II	2 scores<1.5SD or 1 score<2SD
**Visual memory**	WMS III – Visual reproduction I and II	2 scores<1.5SD or 1 score<2SD
**Working memory**	WMS III– Digit span	score <2SD
**Processing speed**	WAIS III – Digit – Symbol – Coding and Symbol search<br>Trail Making Test, part A<br>Stroop test – word reading	At least 3 scores<1SD or 2 scores<1.5SD
**Executive functions**	Stroop test (color naming and word color naming)<br>Trail Making Test, part B and B-A<br>Phonemic Fluency – letters M, R and P<br>Phonemic Fluency – categories of animals<br>18-points Clock drawing test	At least 3 scores<1SD or 2 scores<1.5SD
**Language**	Token Test – short-form	Score <2SD

SD, standard deviation; WAIS III, Wechsler Adult Intelligence Scale Third Edition; WMS III, Wechsler Memory Scale Third Edition.

For peer review only

Figure 1. Identification of potential participants from the lists of COVID-19 test results and hospitalization episodes during the period from March 2020 to February 2021, and from the list of individuals enrolled at primary healthcare units of *Unidade Local de Saúde de Matosinhos* (ULSM). Cohort 1 includes individuals with a positive diagnosis test for COVID-19 registered at ULSM and hospitalized at *Hospital Pedro Hispano* and those with a positive test performed at CHEDV and hospitalized at one of the three hospitals of CHEDV. Cohort 2 includes individuals with no positive diagnosis test for COVID-19 and hospitalized at Hospital Pedro Hispano and at CHEDV. Cohort 3 includes individuals with a positive diagnosis test for COVID-19 registered at ULSM and with clinical follow-up in the community. Cohort 4 includes individuals enrolled at ULSM with no positive diagnosis test for COVID-19 and without any hospitalization episode between March 2020 and February 2021.
Note: Areas are not proportional to the number of cases.

For peer review only

For peer review only - http://bmjopen.bmj.com/site/about/guidelines.xhtml

References

1. Statement on the fourteenth meeting of the International Health Regulations (2005) Emergency Committee regarding the coronavirus disease (COVID-19) pandemic [press release]. 2023.
2. Soriano JB, Murthy S, Marshall JC, Relan P, Diaz JV. A clinical case definition of post-COVID-19 condition by a Delphi consensus. The Lancet Infectious Diseases. 2022;22(4):e102-e7.
3. O'Mahoney LL, Routen A, Gillies C, Ekezie W, Welford A, Zhang A, et al. The prevalence and long-term health effects of Long Covid among hospitalised and non-hospitalised populations: a systematic review and meta-analysis. eClinicalMedicine. 2023;55.
4. Chen C, Haupert SR, Zimmermann L, Shi X, Fritsche LG, Mukherjee B. Global Prevalence of Post-Coronavirus Disease 2019 (COVID-19) Condition or Long COVID: A Meta-Analysis and Systematic Review The Journal of Infectious Diseases. 2022;226(9):1593-607.
5. Delgado-Alonso C, Cuevas C, Oliver-Mas S, Díez-Cirarda M, Delgado-Álvarez A, Gil-Moreno MJ, et al. Fatigue and Cognitive Dysfunction Are Associated with Occupational Status in Post-COVID Syndrome. International Journal of Environmental Research and Public Health. 2022;19(20):13368.
6. Misra S, Kolappa K, Prasad M, Radhakrishnan D, Thakur KT, Solomon T, et al. Frequency of Neurologic Manifestations in COVID-19. A Systematic Review and Meta-analysis. 2021;97(23):e2269-e81.
7. Rao AR, Hidayathullah SM, Hegde K, Adhikari P. Parkinsonism: An emerging post COVID sequelae. IDCases. 2022;27:e01388.
8. Makhoul K, Jankovic J. Parkinson's disease after COVID-19. Journal of the Neurological Sciences. 2021;422.
9. Méndez-Guerrero A, Laespada-García MI, Gómez-Grande A, Ruiz-Ortiz M, Blanco-Palmero VA, Azcarate-Diaz FJ, et al. Acute hypokinetic-rigid syndrome following SARS-CoV-2 infection. Neurology. 2020;95(15):e2109-e18.
10. Cohen ME, Eichel R, Steiner-Birmanns B, Janah A, Ioshpa M, Bar-Shalom R, et al. A case of probable Parkinson's disease after SARS-CoV-2 infection. The Lancet Neurology. 2020;19(10):804-5.
11. Taquet M, Geddes JR, Husain M, Luciano S, Harrison PJ. 6-month neurological and psychiatric outcomes in 236 379 survivors of COVID-19: a retrospective cohort study using electronic health records. The Lancet Psychiatry. 2021;8(5):416-27.
12. Lu Y, Li X, Geng D, Mei N, Wu P-Y, Huang C-C, et al. Cerebral Micro-Structural Changes in COVID-19 Patients – An MRI-based 3-month Follow-up Study. EClinicalMedicine. 2020;25:100484.
13. Bauer L, Laksono BM, de Vrij FMS, Kushner SA, Harschnitz O, van Riel D. The neuroinvasiveness, neurotropism, and neurovirulence of SARS-CoV-2. Trends in Neurosciences. 2022;45(5):358-68.
14. Postuma RB, Aarsland D, Barone P, Burn DJ, Hawkes CH, Oertel W, et al. Identifying prodromal Parkinson's disease: Pre-Motor disorders in Parkinson's disease. Movement Disorders. 2012;27(5):617-26.
15. Postuma RB, Berg D. Advances in markers of prodromal Parkinson disease. Nature Reviews Neurology. 2016;12(11):622-34.
16. Grande G, Rizzuto D, Vetrano DL, Marseglia A, Vanacore N, Laukka EJ, et al. Cognitive and physical markers of prodromal dementia: A 12-year-long population study. Alzheimer's & Dementia. 2020;16(1):153-61.
17. Grande G, Triolo F, Nuara A, Welmer A-K, Fratiglioni L, Vetrano DL. Measuring gait speed to better identify prodromal dementia. Experimental Gerontology. 2019;124:110625.
18. Beauchet O, Annweiler C, Callisaya ML, De Cock A-M, Helbostad JL, Kressig RW, et al. Poor Gait Performance and Prediction of Dementia: Results From a Meta-Analysis. Journal of the American Medical Directors Association. 2016;17(6):482-90.

For peer review only - http://bmjopen.bmj.com/site/about/guidelines.xhtml

19. Araújo N, Severo M, Lopes-Conceição L, Fontes F, Dias T, Branco M, et al. Trajectories of cognitive performance over five years in a prospective cohort of patients with breast cancer (NEON-BC). Breast. 2021;58:130-7.
20. Bauer L, Rissmann M, Benavides FFW, Leijten L, van Run P, Begeman L, et al. In vitro and in vivo differences in neurovirulence between D614G, Delta And Omicron BA.1 SARS-CoV-2 variants. Acta Neuropathologica Communications. 2022;10(1):124.
21. DGS - Direção Geral da Saúde. COVID-19 Relatório de Situação nº 364 de 01-03-2021. https://covid19.min-saude.pt/relatorio-de-situacao/ March 01 2021. Contract No.: 364.
22. Direção Geral da Saúde. Relatório de vacinção-semana 8. República Portuguesa; 2022.
23. Norma 019/2020 Estratégia Nacional de Testes para SARS-CoV-2, (2020).
24. Nasreddine ZS, Phillips NA, Bedirian V, Charbonneau S, Whitehead V, Collin I, et al. The Montreal Cognitive Assessment, MoCA: a brief screening tool for mild cognitive impairment. Journal of the American Geriatrics Society. 2005;53(4):695-9.
25. Freitas S, Simões MR, Martins C, Vilar M, Santana I. Estudos de adaptação do Montreal Cognitive Assessment (MoCA) para a população Portuguesa. Avaliação Psicológica. 2010;9:345-57.
26. Freitas S, Simoes MR, Alves L, Santana I. Montreal Cognitive Assessment (MoCA): normative study for the Portuguese population. Journal of clinical and experimental neuropsychology. 2011;33(9):989-96.
27. Ingraham LJ, Aiken CB. An empirical approach to determining criteria for abnormality in test batteries with multiple measures. Neuropsychology. 1996;10(1):120.
28. Ruano L, Sousa A, Severo M, Alves I, Colunas M, Barreto R, et al. Development of a self-administered web-based test for longitudinal cognitive assessment. Scientific reports. 2016;6:19114.
29. Ruano L, Severo M, Sousa A, Ruano C, Branco M, Barreto R, et al. Tracking cognitive performance in the general population and in patients with mild cognitive impairment with a self-applied computerized test (Brain on Track). Journal of Alzheimer's Disease. 2019;71(2):541-8.
30. Postuma RB, Arnulf I, Hogl B, Iranzo A, Miyamoto T, Dauvilliers Y, et al. A single-question screen for rapid eye movement sleep behavior disorder: A multicenter validation study. Movement Disorders. 2012;27(7):913-6.
31. Postuma RB, Bertrand J-A, Montplaisir J, Desjardins C, Vendette M, Rios Romenets S, et al. Rapid eye movement sleep behavior disorder and risk of dementia in Parkinson's disease: A prospective study. Movement Disorders. 2012;27(6):720-6.
32. Iranzo A, Fernández-Arcos A, Tolosa E, Serradell M, Molinuevo JL, Valldeoriola F, et al. Neurodegenerative disorder risk in idiopathic REM sleep behavior disorder: study in 174 patients. PloS one. 2014;9(2):e89741.
33. Tanner C, Gilley D, Goetz C, editors. A brief screening questionnaire for parkinsonism. Annals of neurology; 1990: LITTLE BROWN CO 34 BEACON STREET, BOSTON, MA 02108-1493.
34. Dahodwala N, Siderowf A, Baumgarten M, Abrams A, Karlawish J. Screening questionnaires for parkinsonism: a systematic review. Parkinsonism Relat Disord. 2012;18(3):216-24.
35. Podsiadlo D, Richardson S. The timed "Up & Go": a test of basic functional mobility for frail elderly persons. Journal of the American Geriatrics Society. 1991;39(2):142-8.
36. Zigmond AS, Snaith RP. The hospital anxiety and depression scale. Acta psychiatrica scandinavica. 1983;67(6):361-70.
37. Pais-Ribeiro J, Silva I, Ferreira T, Martins A, Meneses R, Baltar M. Validation study of a Portuguese version of the Hospital Anxiety and Depression Scale. Psychology, health & medicine. 2007;12(2):225-37.
38. Bjelland I, Dahl AA, Haug TT, Neckelmann D. The validity of the Hospital Anxiety and Depression Scale: An updated literature review. Journal of Psychosomatic Research. 2002;52(2):69-77.

For peer review only - http://bmjopen.bmj.com/site/about/guidelines.xhtml

39. Buysse DJ, Reynolds CF, Monk TH, Berman SR, Kupfer DJ. The Pittsburgh sleep quality index: A new instrument for psychiatric practice and research. Psychiatry Research. 1989;28(2):193-213.
40. Gomes AA, Marques DR, Meiavia AM, Cunha F, Clemente V. Psychometric properties and accuracy of the European Portuguese version of the Pittsburgh Sleep Quality Index in clinical and non-clinical samples. Sleep and Biological Rhythms. 2018;16(4):413-22.
41. Chung F, Yegneswaran B, Liao P, Chung Sharon A, Vairavanathan S, Islam S, et al. STOP Questionnaire: A Tool to Screen Patients for Obstructive Sleep Apnea. Anesthesiology. 2008;108(5):812-21.
42. Reis R, Teixeira F, Martins V, Sousa L, Batata L, Santos C, et al. Validation of a Portuguese version of the STOP-Bang questionnaire as a screening tool for obstructive sleep apnea: Analysis in a sleep clinic. Rev Port Pneumol (2006). 2015;21(2):61-8.
43. Rebelo-Marques A, Vicente C, Valentim B, Agostinho M, Pereira R, Teixeira MF, et al. STOP-Bang questionnaire: the validation of a Portuguese version as a screening tool for obstructive sleep apnea (OSA) in primary care. Sleep Breath. 2018;22(3):757-65.
44. Kalbfleisch J, Prentice R. The statistical analysis of failure time data. 2nd ed. New York, NY: John Wiley & Sons; 2002.
45. Schmand B, Jonker C, Hooijer C, Lindeboom J. Subjective memory complaints may announce dementia. Neurology. 1996;46(1):121-5.
46. Gino S, Mendes T, Maroco J, Ribeiro F, Schmand BA, de Mendonca A, et al. Memory complaints are frequent but qualitatively different in young and elderly healthy people. Gerontology. 2010;56(3):272-7.
47. Borkowski JG, Benton AL, Spreen O. Word fluency and brain damage. Neuropsychologia. 1967;5(2):135-40.
48. Cavaco S, Goncalves A, Pinto C, Almeida E, Gomes F, Moreira I, et al. Semantic fluency and phonemic fluency: regression-based norms for the Portuguese population. Archives of clinical neuropsychology : the official journal of the National Academy of Neuropsychologists. 2013;28(3):262-71.
49. Babins L, Slater ME, Whitehead V, Chertkow H. Can an 18-point clock-drawing scoring system predict dementia in elderly individuals with mild cognitive impairment? Journal of clinical and experimental neuropsychology. 2008;30(2):173-86.
50. Santana I, Duro D, Freitas S, Alves L, Simoes MR. The Clock Drawing Test: Portuguese norms, by age and education, for three different scoring systems. Archives of clinical neuropsychology : the official journal of the National Academy of Neuropsychologists. 2013;28(4):375-87.
51. Cavaco S, Goncalves A, Pinto C, Almeida E, Gomes F, Moreira I, et al. Trail Making Test: regression-based norms for the Portuguese population. Archives of clinical neuropsychology : the official journal of the National Academy of Neuropsychologists. 2013;28(2):189-98.
52. Duro D, Freitas S, Alves L, Simões MR, Santana I. O Teste do Desenho do Relógio: influência das variáveis sociodemográficas e de saúde na população portuguesa. Sinapse. 2012;12(1):5-12.
53. Weschler D. Escala de Memória de Weschler – 3ª Edição (WMS-III): Manual técnico. Lisboa, Portugal: CEGOC; 2008.
54. Wechsler D. WMS-III Wechsler Memory Scale-Third Edition. San Antonio, TX: The Psychological Corporation; 1997.
55. Wechsler D. WAIS-III Wechsler Adult Intelligence Scale-Third Edition. San Antonio, TX: The Psychological Corporation; 1997.
56. Weschler D. Escala de Inteligência de Weschler para Adultos – 3ª Edição (WAIS-III): Manual técnico. Lisboa, Portugal: CEGOC; 2008.
57. Fernandes S. Stroop: Teste de Cores e Palavras. Lisboa, Portugal: CEGOC; 2013.
58. Golden C. Stroop Colour and Word Test: a manual for clinical and experimental uses. Chicago, IL: Soelting; 1978.

For peer review only - http://bmjopen.bmj.com/site/about/guidelines.xhtml

59. Spellacy FJ, Spreen O. A short form of the Token Test. Cortex. 1969;5(4):390-7.
60. Alves L, Simoes MR, Martins C. The estimation of premorbid intelligence levels among Portuguese speakers: the Irregular Word Reading Test (TeLPI). Archives of clinical neuropsychology : the official journal of the National Academy of Neuropsychologists. 2012;27(1):58-68.
61. Beck AT, Steer RA, Brown GK. BDI-II Manual. San Antonio, TX: The Psychological Corporation; 1996.
62. Campos RC, Gonçalves B. The Portuguese version of the Beck Depression Inventory-II (BDI-II): Preliminary psychometric data with two nonclinical samples. European Journal of Psychological Assessment. 2011;27(4):258-64.
63. Mahoney FI, Barthel DW. Funcional evaluation: the Barthel Index. Maryland state medical journal. 1965;14:61-5.
64. Sequeira C. Cuidar de idosos dependentes. Coimbra, Portugal: Quarteto Editora; 2007.
65. Lawton MP, Brody EM. Assessment of older people: self-maintaining and instrumental activities of daily living. The Gerontologist. 1969;9(3):179-86.
66. Madureira S, Moleiro C, Verdelho A, Guerreiro M. Escala de Atividades Instrumentais de Vida Diária (AIVD). In: Simões MR, Santana I, Grupo de Estudos de Envelhecimento Cerebral e Demência, editors. Escalas e Testes na Demência, 3ª Edição. Lisboa, Portugal: Novartis; 2015. p. 140-5.

For peer review only

For peer review only - http://bmjopen.bmj.com/site/about/guidelines.xhtml

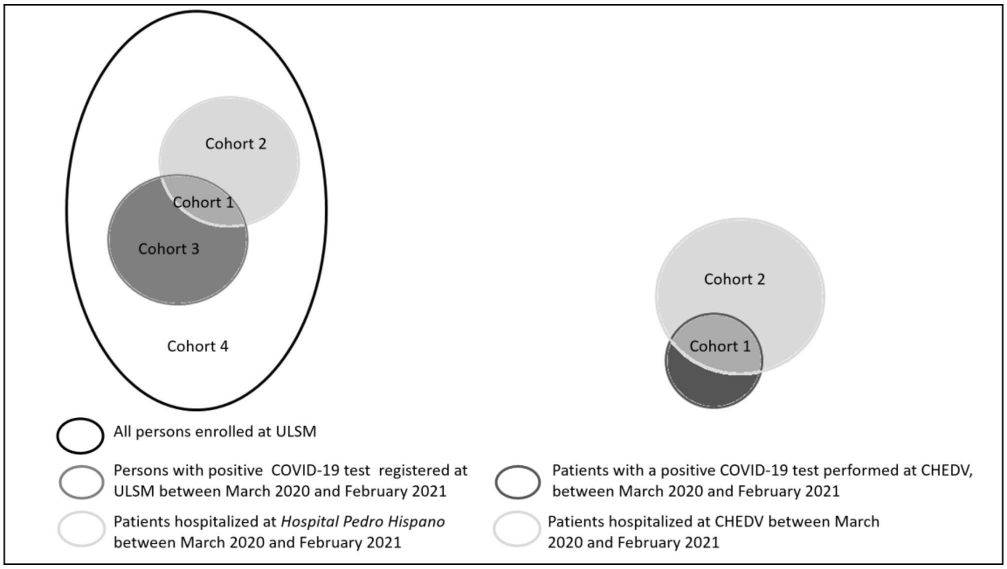

Figure 1. Identification of potential participants from the lists of COVID-19 test results and hospitalization episodes during the period from March 2020 to February 2021, and from the list of individuals enrolled at primary healthcare units of Unidade Local de Saúde de Matosinhos (ULSM). Cohort 1 includes individuals with a positive diagnosis test for COVID-19 registered at ULSM and hospitalized at Hospital Pedro Hispano and those with a positive test performed at CHEDV and hospitalized at one of the three hospitals of CHEDV. Cohort 2 includes individuals with no positive diagnosis test for COVID-19 and hospitalized at Hospital Pedro Hispano and at CHEDV. Cohort 3 includes individuals with a positive diagnosis test for COVID-19 registered at ULSM and with clinical follow-up in the community. Cohort 4 includes individuals enrolled at ULSM with no positive diagnosis test for COVID-19 and without any hospitalization episode between March 2020 and February 2021.
Note: Areas are not proportional to the number of cases.

337x189mm (144 x 144 DPI)

BMJ Open

Long-term neurological complications in COVID-19 survivors: study protocol of a prospective cohort study (NeurodegCoV-19).

Journal:	*BMJ Open*
Manuscript ID	bmjopen-2023-072981.R1
Article Type:	Protocol
Date Submitted by the Author:	03-Jul-2023
Complete List of Authors:	Araujo, Natalia; UP EPIUnit; UP ISPUP ITR<br>Silva, Isa; UP EPIUnit; UP ISPUP ITR<br>Campos, Patrícia; UP EPIUnit; UP ISPUP ITR<br>Correia, Rita; UP EPIUnit; UP ISPUP ITR<br>Calejo, Margarida; ULSM<br>Freitas, Pedro; IPP ESS<br>Seco, Mariana; ULSM<br>Ribeiro, Luís; ULSM<br>Costa, Ana ; University of Porto Epidemiology Research Unit; UP ISPUP ITR<br>Morais, Samantha; University of Porto Epidemiology Research Unit; UP ISPUP ITR<br>Pereira, Susana; University of Porto Epidemiology Research Unit; Francisco Gentil Portuguese Institute for Oncology of Porto, Neurology<br>Firmino-Machado, João; UP EPIUnit, EPIUnit; Universidade de Aveiro, Departamento de Ciências Médicas<br>Rodrigues, Rita; CHEDV<br>Pais, Joana; UP EPIUnit; UP ISPUP ITR<br>Ruano, Luís; UP EPIUnit; CHEDV<br>Lunet, Nuno; FMUP, Department of Clinical Epidemiology, Predictive Medicine and Public Health; UP EPIUnit<br>Tedim-Cruz, Vítor; UP EPIUnit; ULSM
**Primary Subject Heading**:	Epidemiology
Secondary Subject Heading:	Neurology, Mental health, Infectious diseases
Keywords:	COVID-19, Dementia < NEUROLOGY, Neurological injury < NEUROLOGY, Parkinson-s disease < NEUROLOGY, EPIDEMIOLOGIC STUDIES

SCHOLARONE™
Manuscripts

Title

Long-term neurological complications in COVID-19 survivors: study protocol of a prospective cohort study (*NeurodegCoV-19)*.

Natália Araújo[1,2,3], Isa Silva[1,2], Patrícia Campos[1,2], Rita Correia[1,2], Margarida Calejo[4], Pedro Freitas[5], Mariana Seco[4], Luís Ribeiro[4], Rute Costa[1,2,3], Samantha Morais[1,2], Susana Pereira[1,2,6], João Firmino Machado[1,7], Rita Rodrigues[8], Joana Pais[1,2], Luís Ruano[1,2,3,8], Nuno Lunet[1,2,3], Vítor Tedim-Cruz[1,2,4]

[1] EPIUnit – Instituto de Saúde Pública, Universidade do Porto, Rua das Taipas, 135, 4050-600 Porto, Portugal

[2] Laboratório para a Investigação Integrativa e Translacional em Saúde Populacional (ITR), Rua das Taipas, 135, 4050-600 Porto, Portugal

[3] Departamento de Ciências da Saúde Pública e Forenses e Educação Médica, Faculdade de Medicina da Universidade do Porto, Alameda Professor Hernâni Monteiro, 4200-319 Porto, Portugal

[4] Serviço de Neurologia, Unidade Local de Saúde de Matosinhos, 4464-513 Matosinhos, Portugal

[5] Escola Superior de Saúde do Politécnico do Porto, Rua Dr. António Bernardino de Almeida, 400, 4200-072, Porto, Portugal

[6] Instituto Português de Oncologia do Porto, Rua Dr. António Bernardino de Almeida, 4200-072 Porto, Portugal

[7] Departamento de Ciências Médicas, Universidade de Aveiro, 3810-193, Aveiro, Portugal

[8] Serviço de Neurologia, Centro Hospitalar Entre Douro e Vouga, 4520-211 Santa Maria da Feira, Portugal

Corresponding author

Natália Araújo
Rua das Taipas, 135
4050-600 Porto, Portugal
Tel. +351 222 061 820
natalia.araujo@ispup.up.pt

Words count: 4218 (4014 words in the main text: Introduction, Methods and Analysis, Ethics and dissemination)

For peer review only - http://bmjopen.bmj.com/site/about/guidelines.xhtml

Abstract

Background

Evidence suggests an association between SARS-CoV-2 infection and worse performance on cognitive tests, and a higher risk of Parkinson's disease (PD) and dementia up to six and 12 months after infection, respectively. Longer follow-ups with comparison groups are needed to clarify the potentially increased risk of neurodegenerative diseases in COVID-19 survivors, namely those infected before mass vaccination.

Methods

A prospective study started in July 2022 with four cohorts of 150 individuals each, defined according to SARS-CoV-2 infection and hospitalization status between March 2020 and February 2021: cohort 1—hospitalized due to SARS-CoV-2 infection; cohort 2—hospitalized, COVID-19-free; cohort 3—infected, not hospitalized; cohort 4—not infected, not hospitalized. Cohort 2 will be matched to cohort 1 according to age, sex, level of hospitalization care, and length of stay; cohort 4 will be age- and sex-matched to cohort 3. Baseline, one- and two-year follow-up evaluations will include: cognitive performance assessed with the Montreal Cognitive Assessment (MoCA) and neuropsychological tests; the assessment of prodromal markers of PD with Rapid Eye Movement Sleep Behavior Disorder single-question Screen and self-reported olfactory and gustative alterations; screening of PD with the 9-item PD screening questionnaire; gait evaluation with Timed Up&Go test. Suspected cases of cognitive impairment and PD will undergo a clinical evaluation by a neurologist. Frequency measures of neurological complications, prodromal markers, and diagnoses of dementia and PD, will be presented. The occurrence of cognitive decline— the difference between baseline and one-year MoCA scores below 1.5 standard deviations below the mean of the distribution of the variation— will be compared between cohorts 1 and 2, and cohorts 3 and 4 with Odds Ratio estimated using multivariate logistic regression.

Ethics and dissemination

This study received ethics approval from the Ethics Committees of the health units *Unidade Local de Saúde de Matosinhos* and *Centro Hospitalar de Entre Douro e Vouga*, and informed consent is signed for participating. Results will be disseminated among the scientific community and the public.

For peer review only

Strengths and limitations of this study

- Prodromal markers of Parkinson's disease and dementia, as well as diagnoses of these diseases, will be evaluated with screening tests, neuropsychological tests, and a clinical evaluation by a neurologist.
- In addition to assessing episodic cognitive impairment two to three years after SARS-CoV-2 infection, we will evaluate cognitive performance over two years and compare individuals who had mild/severe SARS-CoV-2 infections with the general population or a group of hospitalized patients.
- Data on cognitive performance will only be obtained after SARS-CoV-2 infection, limiting the assessment of causality between SARS-CoV-2 infection and cognitive impairment.
- Sample size, calculated to detect an association between SARS-CoV-2 infection and cognitive decline defined based on the variation in MoCA score over one year, may not be large enough to identify a significant association between COVID-19 and dementia or PD, requiring a longer follow-up and/or external collaborations.
- Misclassification of the exposure in the comparison groups and the possibility of infection after vaccination, may lead to a possibly weaker association than if comparison groups had been completely COVID-19 free over all the study period.

Keywords

COVID-19; Dementia; Neurological injury; Parkinson's disease; Epidemiologic studies

For peer review only

For peer review only - http://bmjopen.bmj.com/site/about/guidelines.xhtml

Introduction

COVID-19 mortality is still high in some countries and the response to the pandemic is not uniform around the world. Mass vaccination, booster vaccine doses offered to the most vulnerable population subgroups, access to adequate treatment of COVID-19, and less aggressive variants have allowed many countries to face new cases of SARS-CoV-2 infection with a lower load on the health system (1). However, the long-term health consequences of SARS-CoV-2 or long COVID may represent an important health problem. Long COVID is defined by the World Health Organization as the continuation or development of new symptoms three months after the initial SARS-CoV-2 infection, with these symptoms lasting for at least two months with no other explanation (2). It may affect 45% of hospitalized and non-hospitalized SARS-CoV-2 infected individuals within four months post-infection (3), and its worldwide prevalence at 120 days from infection, was estimated at 0.49% (higher prevalence among hospitalized patients: 0.54% *vs.* 0.34%), accounting for nearly 38 million cases(4). Fatigue and cognitive complaints are the most frequent symptoms of long COVID (4) and were associated with difficulties in returning to full-time employment (5).

Although COVID-19 is a respiratory disease, neurological manifestations during the acute phase of SARS-CoV-2 infection are common (6). A few cases of parkinsonism developing between five and 32 days after initial symptoms of SARS-CoV-2 infection were described, suggesting parkinsonism as a consequence of mild and severe COVID-19 infection (7, 8, 9, 10, 11). In a large retrospective study (January to December 2020), COVID-19 was also associated with an increased risk of neurologic conditions during the six months following infection, namely, parkinsonism (increased risk in 45%) and dementia (increased risk in 71%), when compared to matched controls of individuals infected with other respiratory tract infections, although the incidence of parkinsonism and dementia were low: 0.11% and 0.67%, respectively, at six months after infection with SARS-CoV-2 (12). A four-point decrease in the Montreal Cognitive Assessment (MoCA) was observed in 21% and 2% of seropositive symptomatic and seronegative asymptomatic individuals for SARS-CoV-2 infection, respectively, from a pre- to a post-pandemic evaluation (253.4 person-years of follow-up) (13), and a cumulative incidence of cognitive impairment (assessed with a telephone interview for cognitive status) of 12.45% was reported 12 months after hospital discharge in a large study in Wuhan, China (14). Neurological symptoms after infection have been corroborated with brain microstructural changes on imaging exams, namely in the central olfactory system, three months after recovery from COVID-19, with no differences according to COVID-19 severity (15). Indeed, there is evidence from *in vivo* (humans),

For peer review only

For peer review only - http://bmjopen.bmj.com/site/about/guidelines.xhtml

in vitro, and animal studies, that the novel coronavirus has the potential to be neuroinvasive (16).

Early detection of neurodegenerative complications during prodromal phases of disease is essential for implementing disease modifying interventions. Impaired olfaction and rapid eye movement (REM) sleep behavior disorder (RBD) are prodromal markers with the highest sensitivity and specificity, respectively, to predict Parkinson's disease (PD) (17, 18). Non-dementia cognitive impairment and low gait speed can be used to predict dementia (19, 20, 21). Moreover, cognitive decline over one year, that is, a deterioration in cognitive performance over this period, may help in identifying individuals who have normal cognitive performance but who will develop a long-term declining trajectory of cognitive performance (22). Therefore, this study protocol describes the ongoing prospective cohort study *NeurodegCoV-19* which aims to:

1) Quantify the association between SARS-CoV-2 infection and one-year cognitive decline, comparing individuals infected with SARS-CoV-2 virus (hospitalized patients or cases with clinical follow-up in the community) and a control group (hospitalized patients or the general population);
2) Describe trajectories of cognitive performance over two years;
3) Estimate frequency measures of the occurrence and persistence of long-term neurologic symptoms and diagnoses, and prodromal markers of PD, in COVID-19 survivors.
4) Explore the associations between SARS-CoV-2 infection and prodromal markers of PD.

In this study, the reference period to define SARS-CoV-2 infection/COVID-19 hospitalization was the first year of the pandemic. Infections in this period are more likely to be associated with an increased risk of neurological complications because vaccines were essentially unavailable and because in vitro and animal models have shown the lower neurotropism and neurovirulence of the Delta and Omicron BA.1 variants (23). Recruitment began on July 01, 2022 and is expected to end in July 2023. Therefore, the baseline evaluation is being performed two to three years after SARS-CoV-2 infection, and the two-year follow-up is expected to be complete in July 2025.

For peer review only - http://bmjopen.bmj.com/site/about/guidelines.xhtml

Methods and Analysis

Setting

This prospective cohort study is based on the population of Matosinhos, one of the counties of the Porto Metropolitan Area, Northern Littoral of Portugal, the region with the highest number of COVID-19 cases during the first months of the pandemic (326 378 cases until February 28, 2021) (24). Nearly all dwellers in Portugal are enrolled in public primary healthcare units, either as regular users, sporadic users or whether they have never used this service, and receive a unique identification number in the National Health System. Those enrolled at these units of Matosinhos (176 299 inhabitants enrolled in 2020, and 177 715 in 2021) were the source population for this study. The local health unit of Matosinhos, *Unidade Local de Saúde de Matosinhos* (ULSM), integrates primary and continuous palliative care units, and its hospital, *Hospital Pedro Hispano,* under a single entity and the same management. The clinical pathology laboratory of ULSM performed all COVID-19 diagnostic tests prescribed by family doctors and public health services of the ULSM, except when results could not be obtained in 24 hours due to an overload of testing; private laboratories collaborated in the latter cases.

Additionally, the hospital unit *Centro Hospitalar de Entre Douro e Vouga (CHEDV)* in *Santa Maria da Feira*, another county of the Porto Metropolitan Area, which includes three hospitals—*Hospital de São Sebastião, E.P.E.* (in *Santa Maria da Feira*), *Hospital Distrital de São João da Madeira*, and *Hospital São Miguel* (in *Oliveira de Azeméis*)— also participated for the selection of hospitalized participants. CHEDV does not include primary health care nor continuous palliative care units.

Study design

This is a prospective study with a baseline assessment two to three years after SARS-CoV-2 infection and follow-up evaluations one and two years later. Baseline assessments started on July 1, 2022, and are expected to end on July, 2023. Participants were retrospectively identified from administrative lists of ULSM and CHEDV, and according to the exposure definition, as follows: having a first diagnosis of SARS-CoV-2 infection (detection of RNA virus in nasopharyngeal or oropharyngeal swabs, with Real-Time Polymerase Chain Reaction), before any vaccination, during the period from March, 2020 (first case diagnosed in Portugal on March 2) to February 2021 [complete vaccination uptake reached 3% and partial vaccination, 6%, on February 28, 2021 (25)]. Exposed individuals are grouped according to hospitalization at *Hospital Pedro Hispano* or at one of the three hospitals of CHEDV due to infection with COVID-19 —

For peer review only - http://bmjopen.bmj.com/site/about/guidelines.xhtml

cohort 1, or clinical follow-up in the community — cohort 3. The unexposed group is defined as having no diagnosis of SARS-CoV-2 infection in the same period, nor after if unvaccinated, and with at least the primary scheme of vaccination, to avoid the inclusion of controls who could get infected during the follow-up period without being vaccinated. Unexposed individuals were also grouped according to having been hospitalized at *Hospital Pedro Hispano* or at CHEDV during the same period— cohort 2, or not — cohort 4.

According to instructions from the Ministry of Health, from October 27, 2020, all patients had to be tested for SARS-CoV-2 infection before hospital admission (26). Therefore, efforts were initially made to select participants infected and/or hospitalized after this date to ensure the non-infection status of the hospitalized comparison group. However, due to a low number of eligible participants for cohort 1, we extended the period to March 2020.

Misclassification regarding the non-exposed comparison groups cannot be excluded as the seronegativity for SARS-CoV-2 infection of the comparison groups could not be confirmed due to the retrospective identification of the participants. Although the National Serological Survey reported a low seroprevalence (2.9%) in May-June 2020 (27), it was higher in February-March 2021 (13.5% due to natural infection) (28).

<u>Participants</u>

Eligible participants are 18 or older at the COVID-19 diagnosis (similar age at baseline for the comparison groups). General and cohort-specific eligibility criteria are checked by consultation of medical records and complemented with information from the participant during recruitment.

<u>SARS-CoV-2 infected patients</u>

Participants with a positive test for SARS-CoV-2 infection between March 2020 and February 2021, were retrospectively identified from the ULSM and CHEDV databases, which, in the case of ULSM, includes tests sampled at the ULSM (96.7%), at temporary emergency units outside the hospital (0.3%,) and the drive-in community center (3.1%), whereas all tests from CHEDV were sampled at the hospitals.

Individuals infected with SARS-CoV-2 were included into one of two groups: cohort 1, individuals hospitalized at *Hospital Pedro Hispano* or at CHEDV due to COVID-19 infection, and cohort 3, individuals with clinical follow-up of COVID-19 in the community (asymptomatic or not). Individuals for cohort 1 were identified in two steps: 1) exclusion, from the list of hospitalization episodes at *Hospital Pedro Hispano* or at CHEDV, of those with a description of Homogeneous

For peer review only - http://bmjopen.bmj.com/site/about/guidelines.xhtml

Diagnostic Groups surely not compatible with virus infection (e.g., childbirth); 2) consultation of medical records of hospitalization episodes not excluded in the previous step, to identify if hospital admission was due to COVID-19, the highest level of care (general ward, intermediate care, intensive care unit), and length of stay (Figure 1). All individuals identified were contacted for recruitment.

Individuals for cohort 3 were those on the ULSM list of positive tests for SARS-CoV-2 infection and not on the ULSM list of hospitalized patients (criterion confirmed by the participant) (Figure 1). Three pools of individuals were selected from the pandemic's first, second, and third waves (earliest 30 diagnosis dates in 2020; all cases from October 27 to November 03, 2020, and the latest 30 diagnosis dates in February 2021).

Unexposed comparison groups

A comparison group of hospitalized patients (cohort 2), with no positive test for SARS-CoV-2 infection between March 2020 and February 2021, never tested positive afterwards or infected after complete primary vaccination, was identified from the list of hospitalized patients between March 2020 and February 2021 who were not on the list of positive tests for COVID-19 for this period (Figure 1). Individuals for cohort 2 will be matched with individuals from cohort 1 regarding the hospital (*Hospital Pedro Hispano or* CHEDV), age (18-24, 25-34, 35-44, 45-54, 55-64, 65-74, and ≥75 years), sex, level of hospitalization care (general ward, intermediate care, intensive care unit), number of days of hospitalization (1, 2-7, 8-15, 16-30, and >30 days), and month of hospital admission.

For comparison with cohort 3, cohort 4 will include (baseline) age- and sex-matched individuals randomly selected from the pool of individuals enrolled at ULSM, who were not on the list of a positive diagnostic test for COVID-19 between March 2020 and February 2021, nor on the list of hospitalized patients in the same period (Figure 1).

Exclusion criteria

We will exclude: 1) individuals diagnosed with a condition impairing cognitive function or enrolled in treatments for cognitive decline (consumption of Memantine, Rivastigmine, or Donepezil; participation in psychotherapeutic interventions for clinical or research purposes), or under clinical neuropsychological assessment, or with a history of substance abuse (registered in the medical file or spontaneously reported by the participant); 2) individuals with less than one year of formal education, and primary education (grades one to four) not in the Portuguese

For peer review only - http://bmjopen.bmj.com/site/about/guidelines.xhtml

education system (criteria for the use of cognitive tests); 3) individuals with a motor, visual or auditory problem incompatible with the performance of the cognitive tests.

Individuals living at senior homes are excluded, as well as those infected between the two doses of the primary vaccination scheme or within the 14 days following vaccination.

The report from the participant of an infection with SARS-CoV-2 virus during the period from March 2020 to February 2021 that could not be confirmed with a registry of a positive result at the RT-PCR test, or a hospitalization episode in a private hospital reported by the participant during this period, will lead to the exclusion of the participant.

<u>Recruitment</u>

Phone calls to contact the participants are performed during the day (morning, afternoon and evening) and with repeated attempts, in case of failure. A trained member of the research team conducts a structured conversation aiming to confirm the eligibility of participants, explain the objectives and procedures of the study, and schedule the in-person evaluation at *Hospital Pedro Hispano* or *Hospital São Sebastião*. Reasons for not participating (impossible to contact, exclusion criteria or refusals) are registered for all potential participants identified for the cohorts 1 to 4.

<u>Baseline and follow-up evaluations</u>

Participants are evaluated in person, at *Hospital Pedro Hispano* or *Hospital São Sebastião*, at baseline, that is, approximately two to three years after the index date, which corresponds to the date of the first positive test for SARS-CoV-2 infection, for cohort 3, the date of hospital admission due to acute COVID-19, for cohort 1, and the date of hospital admission due to other reasons, for cohort 2. No index date was attributed to participants of cohort 4 as they were neither infected nor hospitalized during the period March, 2020 to February, 2021.

Follow-up evaluations after one and two years will be similar to the baseline assessment.

<u>Instruments</u>

Table 1 presents the instruments administered to all participants of the four cohorts.

<u>Structured questionnaire</u>

Trained interviewers conduct face-to-face interviews using a structured questionnaire to collect baseline data on: 1) sociodemographic characteristics: age, years of education, employment

For peer review only

status, occupation; 2) lifestyles: alcohol and tobacco consumption; 3) previous medical diagnoses and current medication intake; 4) neurologic symptoms during the first infection and following recovery (cohorts 1 and 3, and individuals from the comparison groups who got infected after vaccination), and current symptoms (all participants); 5) dates of SARS-CoV-2 infection diagnoses (re-infections for cohorts 1 and 3, and infection episodes after complete vaccination, for cohorts 2 and 4); 6) data on vaccination uptake.

Evaluation of cognitive performance and identification of cognitive impairment — Montreal Cognitive Assessment and Brain on Track, neuropsychological evaluation, and clinical evaluation by a neurologist

Cognitive performance of all participants is evaluated with the Montreal Cognitive Assessment (MoCA), version 7.1 (29), translated, adapted and validated in the Portuguese population (30). MoCA is a screening instrument for mild cognitive impairment, with good psychometric characteristics, and high sensitivity (31). It assesses visuoconstructive ability, language, attention, concentration and working memory, executive functions, delayed memory, and orientation. Scores range 0 to 30, with higher values corresponding to better cognitive performance. Interviewers received training and certification from the MoCA Training and Certification Module at www.mocacognition.com, and the Manual of the Portuguese version 7.1 was discussed within the research team to clarify administration procedures and the rating process.

Two outcome measures will be used: cognitive decline and probable cognitive impairment. We will define the former based on the distribution of the MoCA scores variation over time (score at one-year *minus* score at baseline) in the comparator non-exposed group (either cohort 2 or cohort 4), as being a variation in MoCA scores below 1.5 standard deviations (SD) below the mean. For defining probable cognitive impairment, we use age- and education-specific norms (31) and the cut-off point of 1.5 SD below the mean.

Participants with probable cognitive impairment are invited for a neuropsychological assessment, to be scheduled as soon as possible, to confirm cognitive deficits. The neuropsychological evaluation includes neurocognitive tests validated for the Portuguese population, assessing verbal and visual memory, working memory, information processing speed, executive functions, and language. It also includes the assessment of subjective memory complaints, emotional functioning, functional disability, independent living skills, and pre-morbid intelligence (Table 2).

Based on the number of tests used for evaluating each cognitive domain, cut-off points (below 1 SD, 1.5 SD or 2 SD below the mean of age-norms) are applied to identify cognitive deficit (32)

For peer review only

For peer review only - http://bmjopen.bmj.com/site/about/guidelines.xhtml

(Table 3). Participant with cognitive deficit in at least one cognitive domain are considered to have cognitive impairment. These are referred to a clinical consultation with a neurologist of the research team at the hospital, for clinical follow-up according to the best clinical practices in use and a structured protocol of classification of the probable etiology of the cognitive impairment. Brain imaging and laboratory tests will complement the diagnosis whenever necessary.

Additionally, cognitive performance is evaluated with the web-based remote monitoring test Brain on Track. This instrument showed good internal consistency, discriminative ability and reliability (33, 34). It assesses executive functions, short and delayed memories, attention, calculation, visuoconstructive ability, and language. A complete session of tests of Brain on Track takes 24 minutes and includes 11 exercises with fixed duration. The participant is asked to answer to as many items as possible in each exercise, as the score is the number of correct answers. Based on this score and using a predictive model adjusted for age (to be published), a score of risk for cognitive impairment is computed. Participants with a high score of risk will be invited for the neuropsychological assessment.

Eligibility criteria to use Brain on Track are: at least three years of education, access to a computer at home with internet, and sufficient digital literacy to log into the website, or with help from another person but with autonomy for using the mouse and to do the exercises independently.

All participants eligible to use Brain on Track are invited for remote monitoring. The first and second sessions of tests are scheduled with a one-week interval, and the following sessions every three months thereafter.

Identification of prodromal markers and screening for Parkinson's disease

REM Sleep Behavior Disorder Single-Question Screen

We use the REM Sleep Behavior Disorder Single-Question Screen (35) to identify idiopathic rapid eye movement sleep behavior disorder (RBD), which is an important risk factor for PD (36). RBD may be considered a prodromal marker of PD, dementia with Lewy Bodies, and multiple system atrophy, and about 75% of those suffering from RBD develop PD or a parkinsonism within about 10 years (37). This rapid test has high sensitivity, specificity and reliability (35).

The nine-item Parkinson's disease screening questionnaire

We use the nine-item PD screening questionnaire (38) which has a sensitivity and a specificity ranging 61-92% and 29-92%, respectively, to identify possible cases of PD in community settings

For peer review only - http://bmjopen.bmj.com/site/about/guidelines.xhtml

(39). The test includes nine questions to be answered with *Yes* or *No.* The sum of positive answers is the final score of the test which defines the likelihood of having PD: 0–1 (unlikely), 2–4 (possible), ≥ 5 (probable) (39).

Participants screening positive for possible parkinsonism are formally evaluated by a neurologist of the research team, following usual clinical practices.

Timed "Up and Go" test and gait assessment

We use the Timed "Up and Go" Test (TUG) (40) to measure the ability of participants to perform sequential locomotor tasks that incorporate walking and turning. This test was designed to measure mobility in elderly people and could be a useful tool for quantifying locomotor performance in people with PD. Patients are required to stand up from a chair, walk three meters, turn around, walk back to the chair, and sit down again. One training trial is performed under instructions of a trained member of the research team and then two trials are videotaped to allow a trained neurologist of the research team to later evaluate gait and balance. The time, in seconds, necessary for the participant to perform the test is recorded. The two videotaped trials of the TUG test are evaluated using a structured report comprising: 1) four gait-related features (ability to stand up from a chair, walking characteristics, presence of "freezing" walking, posture); 2) the rating of arms swing, asymmetry of shoulder lift, and tremor at rest on gait; 3) and a global gait diagnosis ("normal" or "altered", with parkinsonism and/or osteoarticular and/or vascular and/or others features).

Assessment of anxiety, depression, sleep quality, and obstructive sleep apnea

Levels of anxiety and depression symptoms are measured using the Hospital Anxiety and Depression Scale (HADS) (41) validated in the Portuguese population (42). This self-administered questionnaire includes seven items assessing anxiety symptoms and seven items for depression symptoms, resulting in the HADS-A and HADS-D subscales, respectively, rated with a four-point Likert scale (0 to 3). Scores of 11 or higher in the subscale are indicative of clinically significant symptoms (42). HADS was designed to be used in patients with physical illness, and for this reason, the questionnaire does not include somatic symptoms, namely insomnia, loss of appetite and fatigue, to avoid overlapping of symptoms due to depression and due to physical conditions. Several studies have shown its suitability in both clinical and community settings (43).

To assess sleep quality, we use the self-administered questionnaire Pittsburgh Sleep Quality Index (44), validated in the Portuguese population (45). This instrument presents 19 items

For peer review only - http://bmjopen.bmj.com/site/about/guidelines.xhtml

related to seven components — subjective sleep quality; sleep latency; sleep duration; habitual sleep efficiency; sleep disturbances; use of sleeping medication; day-time dysfunction. Patients are classified as poor or good sleepers (score equal or higher *vs.* lower than five, respectively).

We use the STOP-Bang questionnaire (46) translated and validated in the Portuguese population (47, 48) to screen obstructive sleep apnea. This test includes eight questions of *Yes* or *No* answer. Three or more positive answers are indicative of obstructive sleep apnea with a sensitivity of 98.6%, and eight positive answers suggest a severe condition, with a sensitivity of 80%, according to estimates obtained in a primary care setting (48). The STOP-Bang questionnaire will also be used to control for the presence of obstructive sleep apnea as mimic of RBD.

For peer review only

Clinical information

Physicians of the research team are collecting clinical information related to the hospitalization episode in a standardized questionnaire, including treatment (oxygen therapy, non-invasive ventilation, orotracheal intubation or invasive ventilation, vasopressors and extracardiac membranous oxygenation), pharmacological treatments for COVID-19, blood and cerebrospinal fluid test results, symptoms, Modified Rankin Scale for Neurological Disability and immunosuppression status, obesity status, comorbidities and medications on admission. This information will be used to classify the hospitalization episode according to the World Health Organization (WHO) Clinical Progression Scale (49) and to the three-risk categories of degrees of inflammation (50).

Data analyses and sample size

Outcome measures

We will report on the following outcomes: neurologic symptoms; prodromal markers of PD; probable cognitive impairment detected with the MoCA; cognitive decline detected with the MoCA; impairment in cognitive domains and overall cognitive impairment identified with the neuropsychological assessment; cognitive impairment and PD diagnoses by neurologist.

Frequency measures of the above-mentioned outcomes will be estimated: prevalence at baseline, and after one and two years; period prevalence over one and two years, and cumulative incidence, which will be estimated at one and two years, considering, death as a competitive event, according to the Kalbfleisch and Prentice method (51).

For peer review only - http://bmjopen.bmj.com/site/about/guidelines.xhtml

Associations between SARS-CoV-2 infection and cognitive outcomes will be estimated using Odds Ratios (OR) or Hazard Ratios (HR) with their corresponding 95% confidence interval (95%CI), obtained from multivariate logistic and Cox regressions, respectively, and considering sex, age, education, co-morbidities, and disease severity (not hospitalized, hospitalized in ICU, intermediate or general ward, duration of hospitalization, WHO Clinical Progression Scale, and degrees of inflammation) as co-variables.

The contribution of anxiety and depression symptoms, sleep quality, and obstructive sleep apnea to cognitive decline will also be evaluated using multivariate logistic regression.

Mixed-effect models will be adjusted to compare cognitive trajectories (considering age and education) over two years, according to sociodemographic and clinical characteristics, for each of the cohorts.

Sample size calculation

In order to compare two cohorts of similar disease severity (among hospitalized – cohorts 1 and 2, or among the community – cohorts 3 and 4), considering the occurrence of cognitive decline of 7 per 100 person-years in the non-COVID-19 participants (variation in MoCA scores below 1.5 standard deviation of the normal distribution of the variation), a ratio exposed:unexposed of 1:1, the statistical power of 80% and 5% significance level, 600 participants will be needed (150 in each cohort) to estimate the associations corresponding to a OR of at least 2.5.

This sample size will also be adequate to estimate the frequency of outcomes equal to or lower than 10% with 95% CI, and a margin of error of 7%, as well as incidence rates as low as up to 3.5 per 100 person-years with 95%CI up to 3 per 100 person-years wide.

Considering that individuals of the comparison groups as well as participants of cohorts 1 and 3, may have had SARS-CoV-2 infection episodes more than once after vaccination, including in a short time before baseline and follow-up evaluations, the associations estimated may be weaker than if the comparison groups were completely free from COVID-19. Sensitivity analyses will be performed and an increase in sample size of cohorts 2 and 4 may be needed to increase the proportion of participants without any history of COVID-19.

Patient and Public Involvement Statement

Participants were not involved in the planning of the research.

For peer review only - http://bmjopen.bmj.com/site/about/guidelines.xhtml

Ethics and dissemination

This study received approval from the Ethics Committee of ULSM (117/CES/JAS), and CHEDV (39/2022), and from the ULSM Local Unit of Data Protection and Security (030/CLPSI/2021), as well as from the Data Protection Officer of the *Instituto de Saúde Pública da Universidade do Porto*. A Data Protection Assessment Impact was performed to evaluate and understand the processing, the need and proportionality of data processing. Therefore, this study complies with the World Medical Association Declaration of Helsinki and the General Data Protection Regulation.

Participants are informed about the institutions involved in the project and its research members; the objectives and procedures of the research; data to be collected, namely clinical data from their electronic health record, questionnaires, cognitive tests, and a video; expected individual benefits and risks (minimal); procedures to respect their right to confidentiality, their right and how to manifest this right, to abandon the study and withdraw their consent at any time and that this will not interfere with their medical care, and their right to access their data and have their data corrected and erased. After this explanation, patients clearly manifesting that they do not understand the information will not be considered eligible. Participants will have time to decide. Participation will be assumed after a participant signs the informed consent. No direct or indirect incentives for participation will be given. This is an observational study and no risk is expected. Participants with neurologic complications of COVID-19 will be followed as are non-participants of the study, according to clinical practice implemented at the health institution. Therefore, participants will not be submitted to additional risk.

The findings of this project will be submitted for publication in international peer-reviewed journals, and proposed for presentation in relevant national and international conferences, which will allow for the dissemination of the main findings across the medical community. Press releases through mass media will also be issued to promote the dissemination of information relevant to the general population and policy-makers. Furthermore, the project will contribute to the training of researchers through the production of masters' dissertations and doctoral theses.

Author statement

NA, MC, ARC (Rute Costa), SM, SP, FM, JP, LR, NL, VTC contributed for the conception and design of the work, as well as for obtaining funding. NA, ARC, IS and PC contributed for the design of the structured questionnaire and interview. IS and PC have recruited the participants. NA, IS, PC, and RC (Rita Correia), have performed the general evaluation of participants. PC and RC have

For peer review only - http://bmjopen.bmj.com/site/about/guidelines.xhtml

performed the neuropsychological evaluation. PF has inserted the data in the database. MS has collected clinical data of the participants. MC, RR, LR, VTC has performed the clinical evaluation by neurologist. NA, IS, PC, RC, MC, JP, LR, NL, VTC will analyze and interpret the data. NA, IS, and PC contributed for drafting the work and all authors revised it critically for important intellectual content. All authors approved the final version of the manuscript to be published.

Data statement

The datasets used and/or analyzed during the current study are available from the corresponding author on reasonable request.

Funding

This study is financed by national funds through the FCT - Foundation for Science and Technology, I.P., within the scope of the projects UIDB/04750/2020 and LA/P/0064/2020, and obtained specific FCT funds through the competitive call for Research and Development projects in all scientific domains (PTDC/SAU-EPI/6275/2020).

Competing interests statement

The authors declare that they have no competing interests.

For peer review only

For peer review only - http://bmjopen.bmj.com/site/about/guidelines.xhtml

444 **Table 1.** Description of the instruments used for the evaluation of all participants.

Instrument	Description	Domains/subscales	Score
**MoCA (29)**	Paper and pencil cognitive screening test for evaluating mild cognitive impairment.	Attention and concentration; executive functions; memory; language; visuoconstructional skills; calculations; orientation.	Range: 0 to 30<br>Higher scores represent better cognitive performance.
**18-point CDT (52, 53)**	The Clock-Drawing Test is an assessment tool where participants are asked to draw a big circle, place the clock numbers, and then indicate the time as "10 past 11". An 18-point system will be used to score the test.	Visuospatial; executive function.	Range: 0 to 18<br>Scoring system with three main components: (a) assessment of circle integrity (two points); (b) number placement and sequencing (six points); and (c) placement and size of the hands (six points). Additionally, there are two points for representation of the clock's center and two points for general gestalt.
**Brain on Track (33, 34)**	Computerized and self-administered test that allows longitudinal monitoring of cognitive performance.	Attention; memory; executive functions; language; calculation; constructive ability; visuospatial processing.	Range: virtually unlimited (maximum number of correct answers in a fixed time).<br>Higher scores represent better cognitive performance.<br>Scores falling below an expected performance threshold for each age/education group represent a pattern of decline in individual performance.
**HADS (45)**	Scale with 14 questions designed to assess anxiety and depression. Patients should answer considering the previous week.	Depression; anxiety.	Range (for each subscale): 0 to 21<br>Scores greater than or equal to 11 represent a case of anxiety or depression, as applicable.
**PSQI (44)**	Quality of sleep index with 18 questions about the sleeping habits of the patients during the previous month.	Subjective sleep quality; sleep latency; duration of sleep; habitual sleep efficiency; sleep disorders; use of medications for sleep; daytime dysfunction.	Range: 0 to 21<br>Scores greater than five indicate poor sleep quality.
**REM Sleep Behavior Disorder Single-Question Screen (35)**	Single screening yes/no question about the classic dream-enactment behavior in the REM Behaviour Disorder.	REM sleep behavior disorder.	A positive answer suggests a presence of REM Behaviour Disorder.
**STOP-Bang questionnaire (46)**	Screening tool with eight yes or no questions related to the clinical features of sleep apnea.	Obstructive sleep apnea (OSA).	Range: 0 to 8<br>Each positive answer sum 1 to the final score, with higher scores representing more risk of OSA.<br>Scores equal or greater than three indicate OSA.
**9-item PD screening questionnaire (38)**	Screening tool with nine yes or no questions	PD	Range: 0 to 9<br>Higher scores indicate higher likelihood of Parkinson' Disease: unlikely if scores are 0–1 ; possible if scores are 2–4; and probable if scores are ≥ 5
**Timed "Up and Go" test (40)**	Test to evaluate the ability of patients to perform sequential locomotor tasks that incorporate walking and turning.	Mobility; gait and balance<br>(ability to stand up from the chair;<br>walking characteristics; presence of "freezing" walking; posture)	5-point Likert scale (normal, discreet, mild, moderate, and severe) and global gait diagnosis: normal or altered, with parkinsonism and/or osteoarticular and/or vascular and/or others features

445 MoCA – Montreal Cognitive Assessment; BoT – Brain on Track; HADS – Hospital Anxiety and Depression Scale; PSQI – Pittsburgh Sleep Quality Index.

For peer review only - http://bmjopen.bmj.com/site/about/guidelines.xhtml

Table 2. Description of the battery of instruments used for the neuropsychological evaluation of the participants with probable cognitive impairment*.

Instrument	Description	Cognitive domains/function	Score
**SMC (54, 55)**	A 10-item scale regarding subjective memory complaints.	Subjective memory.	Range: 0 to 21<br>Higher scores reflect maximal memory complaints.
**Verbal Fluency Test (56, 57)**	Verbal fluency tests consist of four trials, one of semantic fluency and three of phonemic fluency, of one minute each. The participants are asked to generate the name of as many species of animals as possible in the semantic fluency test, and as many words as possible beginning with a specific letter in the phonemic fluency test, within one minute.	Non-motor processing speed, language production, and executive functions	The total trial score corresponds to the number of words correctly produced within one minute. The total test score corresponds to the sum of the three trials.<br><br>Higher scores correspond to better performance.
**TMT (58, 59)**	Part A: the participants are asked to draw lines to connect 25 randomly scattered numbered circles in ascending order. They should do so as quickly as possible.<br><br>Part B: the participants are asked to draw lines to connect circles in numeric and alphabetic order, alternating between numbers and letters, progressively up to number 13. They should do so as quickly as possible.	Part A: attention; visual scanning; and speed of eye-hand coordination and information processing.<br>Part B: working memory and executive functions; particularly, the ability to switch between sets of stimuli.	Direct measures of performance: time (seconds) to complete part A and part B and performance errors during part A and part B.<br>Derived scores: difference score (B - A), ratio score (B/A), proportion score (B - A/A), sum score (A + B), and multiplication score (A × B/100).<br>Lower raw scores and higher adjusted scores correspond to better performance.
**WMS-III (60, 61)**	Scale composed of 17 subtests that assess different memory functions using verbal and visual stimuli. Useful in the evaluation of various clinical conditions, including neurodegenerative diseases such as Alzheimer's disease.<br>Subtests used: Logical Memory I, II; Visual Reproduction I, II; Digit Span	Verbal and visual memories; working memory.	Range: Immediate recall and Delayed recall: 0-50; Auditory recognition; Digit span: 0-30; Visual reproduction: 0-104<br>Higher scores correspond to better performance.
**WAIS-III (62, 63)**	Scale composed of 14 subtests that assess the verbal component and the perceptual-motor component of intelligence in adults and older adolescents.<br>Subtests used: Digit-Symbol-Coding; Symbol Search; Information.	Attention/concentration; executive function (sequencing); motor function; processing speed.	The number of correct symbols within the allowed time (120 seconds) is measured.
**Stroop Test (64, 65)**	The Stroop Test evaluates the interference effects between the two cerebral hemispheres. Consist in three trials: 1) word reading; 2) colours naming and 3) identifying the colours of words, without considering the meaning of the word.	Executive functions (inhibitory control); selective attention.	Scores for each trial indicate the number of correct responses. An interference score can be generated that quantifies the participant's ability to inhibit the inappropriate response of reading the colour name as opposed to the colour of the ink used to print the colour name in the third trial.
**Token Test – short form (66)**	A test designed to assess the comprehension of commands that vary in degree of linguistic difficulty. The participant is presented with tokens of different geometric shapes, sizes, and colours, and is required to perform certain acts with the tokens, such as point to tokens, touch them, pick them up, and place one token on top of another.	Attention and vigilance; verbal functions.	Range: 0-36
**TeLPI (67)**	Designed to assess premorbid intelligence, TeLPI is a Portuguese irregular word reading test. The participants are asked to read 46 irregular words, infrequent Portuguese words.	Premorbid Intelligence Quotient (IQ): full scale IQ; Verbal IQ; performance IQ	Range: number of errors (maximum of 46) and years of education are inserted in three linear equations to calculate the three types of IQ
**BDI-II (68, 69)**	A 21-question measure assessing the presence of depressive symptoms experienced by the participant within the past week.	Emotional functioning.	Range: 0 to 63<br>A cut-off score indicative of mild depressive symptoms is greater than 10 and for severe depressive symptoms is greater than 30.
**Barthel ADL Index (70, 71)**	An index to measure functional disability, focused on bodily oriented personal care.	Functional domains: feeding; incontinence; transferring; toileting; dressing; bathing.	Range: 0 to 100<br>Lower scores reflect increased disability.
**IADL (72, 73)**	An eight-item scale used to assess independent living skills which include more complex activities (i.e. "instrumental activities of daily living") necessary for functioning in community settings.	Functional domains: using the telephone; shopping; food preparation; housekeeping; laundry; transport; medication; finances.	Range: 0 to 8<br>Higher scores reflect high function, independence.

Barthel ADL Index – Barthel Activities of Daily Living Index; BDI-II – Beck Depression Inventory – Second Edition; CDT – Clock Drawing Test; IADL – Lawton and Brody Instrumental Activities of Daily Living; SDMT – Symbol and Digit Modalities Test; SMC – Subjective Memory Complains scale; TeLPI – Irregular Word Reading Test; TMT – Trail Making Test; WAIS-III – Wechsler Adult Intelligence Scale – Third Edition; WMS-III – Wechsler Memory Scale – Third Edition.
* Participants with a MoCA score below age- and education-specific values based on norms and the cut-off point of 1.5 standard deviations below the mean

For peer review only

For peer review only - http://bmjopen.bmj.com/site/about/guidelines.xhtml

Table 3. Criteria used for the classification of cognitive impairment, considering the number of tests administered to assess each cognitive domain.

**Cognitive domain**	**Test**	**Criteria for impairment**
**Verbal memory**	WMS III – Logical memory I and II	2 scores<1.5 SD or<br>1 score< 2 SD
**Visual memory**	WMS III – Visual reproduction I and II	2 scores<1.5 SD or<br>1 score< 2 SD
**Attention/Information processing speed**	WAIS III – Digit – Symbol – Coding and Symbol search	At least 3 scores<1 SD or<br>2 scores<1.5 SD
	Trail Making Test, part A	
	Stroop test – word reading	
**Executive functions**	Stroop test (color naming and word color naming)	At least 3 scores<1.5 SD or<br>2 scores<2 SD
	Trail Making Test, part B and B-A	
	Phonemic Fluency – letters M, R and P	
	Phonemic Fluency – categories of animals	
	WMS III– Digit span	
**Language**	Token Test – short-form	Score <2 SD

SD, standard deviation; WAIS III, Wechsler Adult Intelligence Scale Third Edition; WMS III, Wechsler Memory Scale Third Edition.

For peer review only

Figure 1. Identification of potential participants from the lists of COVID-19 test results and hospitalization episodes during the period from March 2020 to February 2021, and from the list of individuals enrolled at primary healthcare units of *Unidade Local de Saúde de Matosinhos* (ULSM). Cohort 1 includes individuals with a positive diagnosis test for COVID-19 registered at ULSM and hospitalized at *Hospital Pedro Hispano* and those with a positive test performed at CHEDV and hospitalized at one of the three hospitals of CHEDV. Cohort 2 includes individuals with no positive diagnosis test for COVID-19 and hospitalized at Hospital Pedro Hispano and at CHEDV. Cohort 3 includes individuals with a positive diagnosis test for COVID-19 registered at ULSM and with clinical follow-up in the community. Cohort 4 includes individuals enrolled at ULSM with no positive diagnosis test for COVID-19 and without any hospitalization episode between March 2020 and February 2021.
Note: Areas are not proportional to the number of cases.

For peer review only

For peer review only - http://bmjopen.bmj.com/site/about/guidelines.xhtml

References

1. Statement on the fourteenth meeting of the International Health Regulations (2005) Emergency Committee regarding the coronavirus disease (COVID-19) pandemic [press release]. 2023.
2. Soriano JB, Murthy S, Marshall JC, Relan P, Diaz JV. A clinical case definition of post-COVID-19 condition by a Delphi consensus. The Lancet Infectious Diseases. 2022;22(4):e102-e7.
3. O'Mahoney LL, Routen A, Gillies C, Ekezie W, Welford A, Zhang A, et al. The prevalence and long-term health effects of Long Covid among hospitalised and non-hospitalised populations: a systematic review and meta-analysis. eClinicalMedicine. 2023;55.
4. Chen C, Haupert SR, Zimmermann L, Shi X, Fritsche LG, Mukherjee B. Global Prevalence of Post-Coronavirus Disease 2019 (COVID-19) Condition or Long COVID: A Meta-Analysis and Systematic Review The Journal of Infectious Diseases. 2022;226(9):1593-607.
5. Delgado-Alonso C, Cuevas C, Oliver-Mas S, Díez-Cirarda M, Delgado-Álvarez A, Gil-Moreno MJ, et al. Fatigue and Cognitive Dysfunction Are Associated with Occupational Status in Post-COVID Syndrome. International Journal of Environmental Research and Public Health. 2022;19(20):13368.
6. Misra S, Kolappa K, Prasad M, Radhakrishnan D, Thakur KT, Solomon T, et al. Frequency of Neurologic Manifestations in COVID-19. A Systematic Review and Meta-analysis. 2021;97(23):e2269-e81.
7. Rao AR, Hidayathullah SM, Hegde K, Adhikari P. Parkinsonism: An emerging post COVID sequelae. IDCases. 2022;27:e01388.
8. Makhoul K, Jankovic J. Parkinson's disease after COVID-19. Journal of the Neurological Sciences. 2021;422.
9. Méndez-Guerrero A, Laespada-García MI, Gómez-Grande A, Ruiz-Ortiz M, Blanco-Palmero VA, Azcarate-Diaz FJ, et al. Acute hypokinetic-rigid syndrome following SARS-CoV-2 infection. Neurology. 2020;95(15):e2109-e18.
10. Cohen ME, Eichel R, Steiner-Birmanns B, Janah A, Ioshpa M, Bar-Shalom R, et al. A case of probable Parkinson's disease after SARS-CoV-2 infection. The Lancet Neurology. 2020;19(10):804-5.
11. Boura I, Chaudhuri KR. Coronavirus Disease 2019 and Related Parkinsonism: The Clinical Evidence Thus Far. Movement Disorders Clinical Practice. 2022;9(5):584-93.
12. Taquet M, Geddes JR, Husain M, Luciano S, Harrison PJ. 6-month neurological and psychiatric outcomes in 236 379 survivors of COVID-19: a retrospective cohort study using electronic health records. The Lancet Psychiatry. 2021;8(5):416-27.
13. Del Brutto OH, Wu S, Mera RM, Costa AF, Recalde BY, Issa NP. Cognitive decline among individuals with history of mild symptomatic SARS-CoV-2 infection: A longitudinal prospective study nested to a population cohort. European Journal of Neurology. 2021;28(10):3245-53.
14. Liu Y-H, Chen Y, Wang Q-H, Wang L-R, Jiang L, Yang Y, et al. One-Year Trajectory of Cognitive Changes in Older Survivors of COVID-19 in Wuhan, China: A Longitudinal Cohort Study. JAMA Neurology. 2022;79(5):509-17.
15. Lu Y, Li X, Geng D, Mei N, Wu P-Y, Huang C-C, et al. Cerebral Micro-Structural Changes in COVID-19 Patients – An MRI-based 3-month Follow-up Study. EClinicalMedicine. 2020;25:100484.
16. Bauer L, Laksono BM, de Vrij FMS, Kushner SA, Harschnitz O, van Riel D. The neuroinvasiveness, neurotropism, and neurovirulence of SARS-CoV-2. Trends in Neurosciences. 2022;45(5):358-68.
17. Postuma RB, Aarsland D, Barone P, Burn DJ, Hawkes CH, Oertel W, et al. Identifying prodromal Parkinson's disease: Pre-Motor disorders in Parkinson's disease. Movement Disorders. 2012;27(5):617-26.
18. Postuma RB, Berg D. Advances in markers of prodromal Parkinson disease. Nature Reviews Neurology. 2016;12(11):622-34.

For peer review only - http://bmjopen.bmj.com/site/about/guidelines.xhtml

19. Grande G, Rizzuto D, Vetrano DL, Marseglia A, Vanacore N, Laukka EJ, et al. Cognitive and physical markers of prodromal dementia: A 12-year-long population study. Alzheimer's & Dementia. 2020;16(1):153-61.

20. Grande G, Triolo F, Nuara A, Welmer A-K, Fratiglioni L, Vetrano DL. Measuring gait speed to better identify prodromal dementia. Experimental Gerontology. 2019;124:110625.

21. Beauchet O, Annweiler C, Callisaya ML, De Cock A-M, Helbostad JL, Kressig RW, et al. Poor Gait Performance and Prediction of Dementia: Results From a Meta-Analysis. Journal of the American Medical Directors Association. 2016;17(6):482-90.

22. Araújo N, Severo M, Lopes-Conceição L, Fontes F, Dias T, Branco M, et al. Trajectories of cognitive performance over five years in a prospective cohort of patients with breast cancer (NEON-BC). Breast. 2021;58:130-7.

23. Bauer L, Rissmann M, Benavides FFW, Leijten L, van Run P, Begeman L, et al. In vitro and in vivo differences in neurovirulence between D614G, Delta And Omicron BA.1 SARS-CoV-2 variants. Acta Neuropathologica Communications. 2022;10(1):124.

24. DGS - Direção Geral da Saúde. COVID-19 Relatório de Situação nº 364 de 01-03-2021. https://covid19.min-saude.pt/relatorio-de-situacao/ March 01 2021. Contract No.: 364.

25. Direção Geral da Saúde. Relatório de vacinção-semana 8. República Portuguesa; 2022.

26. Norma 019/2020 Estratégia Nacional de Testes para SARS-CoV-2, (2020).

27. Kislaya I, Gonçalves P, Barreto M, De Sousa R, Garcia AC, Matos R, et al. Seroprevalence of SARS-CoV-2 infection in Portugal in May-July 2020: results of the first national serological survey (ISNCOVID-19). Acta medica portuguesa. 2021;34(2):87-94.

28. Kislaya I, Gonçalves P, Gómez V, Gaio V, Roquette R, Barreto M, et al. SARS-CoV-2 seroprevalence in Portugal following the third epidemic wave: results of the second National Serological Survey (ISN2COVID-19). Infectious Diseases. 2022;54(6):418-24.

29. Nasreddine ZS, Phillips NA, Bedirian V, Charbonneau S, Whitehead V, Collin I, et al. The Montreal Cognitive Assessment, MoCA: a brief screening tool for mild cognitive impairment. Journal of the American Geriatrics Society. 2005;53(4):695-9.

30. Freitas S, Simões MR, Martins C, Vilar M, Santana I. Estudos de adaptação do Montreal Cognitive Assessment (MoCA) para a população Portuguesa. Avaliação Psicológica. 2010;9:345-57.

31. Freitas S, Simoes MR, Alves L, Santana I. Montreal Cognitive Assessment (MoCA): normative study for the Portuguese population. Journal of clinical and experimental neuropsychology. 2011;33(9):989-96.

32. Ingraham LJ, Aiken CB. An empirical approach to determining criteria for abnormality in test batteries with multiple measures. Neuropsychology. 1996;10(1):120.

33. Ruano L, Sousa A, Severo M, Alves I, Colunas M, Barreto R, et al. Development of a self-administered web-based test for longitudinal cognitive assessment. Scientific reports. 2016;6:19114.

34. Ruano L, Severo M, Sousa A, Ruano C, Branco M, Barreto R, et al. Tracking cognitive performance in the general population and in patients with mild cognitive impairment with a self-applied computerized test (Brain on Track). Journal of Alzheimer's Disease. 2019;71(2):541-8.

35. Postuma RB, Arnulf I, Hogl B, Iranzo A, Miyamoto T, Dauvilliers Y, et al. A single-question screen for rapid eye movement sleep behavior disorder: A multicenter validation study. Movement Disorders. 2012;27(7):913-6.

36. Postuma RB, Bertrand J-A, Montplaisir J, Desjardins C, Vendette M, Rios Romenets S, et al. Rapid eye movement sleep behavior disorder and risk of dementia in Parkinson's disease: A prospective study. Movement Disorders. 2012;27(6):720-6.

37. Iranzo A, Fernández-Arcos A, Tolosa E, Serradell M, Molinuevo JL, Valldeoriola F, et al. Neurodegenerative disorder risk in idiopathic REM sleep behavior disorder: study in 174 patients. PloS one. 2014;9(2):e89741.

For peer review only - http://bmjopen.bmj.com/site/about/guidelines.xhtml

38. Tanner C, Gilley D, Goetz C, editors. A brief screening questionnaire for parkinsonism. Annals of neurology; 1990: LITTLE BROWN CO 34 BEACON STREET, BOSTON, MA 02108-1493.
39. Dahodwala N, Siderowf A, Baumgarten M, Abrams A, Karlawish J. Screening questionnaires for parkinsonism: a systematic review. Parkinsonism Relat Disord. 2012;18(3):216-24.
40. Podsiadlo D, Richardson S. The timed "Up & Go": a test of basic functional mobility for frail elderly persons. Journal of the American Geriatrics Society. 1991;39(2):142-8.
41. Zigmond AS, Snaith RP. The hospital anxiety and depression scale. Acta psychiatrica scandinavica. 1983;67(6):361-70.
42. Pais-Ribeiro J, Silva I, Ferreira T, Martins A, Meneses R, Baltar M. Validation study of a Portuguese version of the Hospital Anxiety and Depression Scale. Psychology, health & medicine. 2007;12(2):225-37.
43. Bjelland I, Dahl AA, Haug TT, Neckelmann D. The validity of the Hospital Anxiety and Depression Scale: An updated literature review. Journal of Psychosomatic Research. 2002;52(2):69-77.
44. Buysse DJ, Reynolds CF, Monk TH, Berman SR, Kupfer DJ. The Pittsburgh sleep quality index: A new instrument for psychiatric practice and research. Psychiatry Research. 1989;28(2):193-213.
45. Gomes AA, Marques DR, Meiavia AM, Cunha F, Clemente V. Psychometric properties and accuracy of the European Portuguese version of the Pittsburgh Sleep Quality Index in clinical and non-clinical samples. Sleep and Biological Rhythms. 2018;16(4):413-22.
46. Chung F, Yegneswaran B, Liao P, Chung Sharon A, Vairavanathan S, Islam S, et al. STOP Questionnaire: A Tool to Screen Patients for Obstructive Sleep Apnea. Anesthesiology. 2008;108(5):812-21.
47. Reis R, Teixeira F, Martins V, Sousa L, Batata L, Santos C, et al. Validation of a Portuguese version of the STOP-Bang questionnaire as a screening tool for obstructive sleep apnea: Analysis in a sleep clinic. Rev Port Pneumol (2006). 2015;21(2):61-8.
48. Rebelo-Marques A, Vicente C, Valentim B, Agostinho M, Pereira R, Teixeira MF, et al. STOP-Bang questionnaire: the validation of a Portuguese version as a screening tool for obstructive sleep apnea (OSA) in primary care. Sleep Breath. 2018;22(3):757-65.
49. Marshall JC, Murthy S, Diaz J, Adhikari NK, Angus DC, Arabi YM, et al. A minimal common outcome measure set for COVID-19 clinical research. The Lancet Infectious Diseases. 2020;20(8):e192-e7.
50. Rubio-Rivas M, Corbella X, Formiga F, Menéndez Fernández E, Martín Escalante MD, Baños Fernández I, et al. Risk Categories in COVID-19 Based on Degrees of Inflammation: Data on More Than 17,000 Patients from the Spanish SEMI-COVID-19 Registry. Journal of Clinical Medicine. 2021;10(10):2214.
51. Kalbfleisch J, Prentice R. The statistical analysis of failure time data. 2nd ed. New York, NY: John Wiley & Sons; 2002.
52. Babins L, Slater ME, Whitehead V, Chertkow H. Can an 18-point clock-drawing scoring system predict dementia in elderly individuals with mild cognitive impairment? Journal of clinical and experimental neuropsychology. 2008;30(2):173-86.
53. Santana I, Duro D, Freitas S, Alves L, Simoes MR. The Clock Drawing Test: Portuguese norms, by age and education, for three different scoring systems. Archives of clinical neuropsychology : the official journal of the National Academy of Neuropsychologists. 2013;28(4):375-87.
54. Schmand B, Jonker C, Hooijer C, Lindeboom J. Subjective memory complaints may announce dementia. Neurology. 1996;46(1):121-5.
55. Gino S, Mendes T, Maroco J, Ribeiro F, Schmand BA, de Mendonca A, et al. Memory complaints are frequent but qualitatively different in young and elderly healthy people. Gerontology. 2010;56(3):272-7.

For peer review only - http://bmjopen.bmj.com/site/about/guidelines.xhtml

56. Borkowski JG, Benton AL, Spreen O. Word fluency and brain damage. Neuropsychologia. 1967;5(2):135-40.

57. Cavaco S, Goncalves A, Pinto C, Almeida E, Gomes F, Moreira I, et al. Semantic fluency and phonemic fluency: regression-based norms for the Portuguese population. Archives of clinical neuropsychology : the official journal of the National Academy of Neuropsychologists. 2013;28(3):262-71.

58. Cavaco S, Goncalves A, Pinto C, Almeida E, Gomes F, Moreira I, et al. Trail Making Test: regression-based norms for the Portuguese population. Archives of clinical neuropsychology : the official journal of the National Academy of Neuropsychologists. 2013;28(2):189-98.

59. Duro D, Freitas S, Alves L, Simões MR, Santana I. O Teste do Desenho do Relógio: influência das variáveis sociodemográficas e de saúde na população portuguesa. Sinapse. 2012;12(1):5-12.

60. Weschler D. Escala de Memória de Weschler – 3ª Edição (WMS-III): Manual técnico. Lisboa, Portugal: CEGOC; 2008.

61. Wechsler D. WMS-III Wechsler Memory Scale-Third Edition. San Antonio, TX: The Psychological Corporation; 1997.

62. Wechsler D. WAIS-III Wechsler Adult Intelligence Scale-Third Edition. San Antonio, TX: The Psychological Corporation; 1997.

63. Weschler D. Escala de Inteligência de Weschler para Adultos – 3ª Edição (WAIS-III): Manual técnico. Lisboa, Portugal: CEGOC; 2008.

64. Fernandes S. Stroop: Teste de Cores e Palavras. Lisboa, Portugal: CEGOC; 2013.

65. Golden C. Stroop Colour and Word Test: a manual for clinical and experimental uses. Chicago, IL: Soelting; 1978.

66. Spellacy FJ, Spreen O. A short form of the Token Test. Cortex. 1969;5(4):390-7.

67. Alves L, Simoes MR, Martins C. The estimation of premorbid intelligence levels among Portuguese speakers: the Irregular Word Reading Test (TeLPI). Archives of clinical neuropsychology : the official journal of the National Academy of Neuropsychologists. 2012;27(1):58-68.

68. Beck AT, Steer RA, Brown GK. BDI-II Manual. San Antonio, TX: The Psychological Corporation; 1996.

69. Campos RC, Gonçalves B. The Portuguese version of the Beck Depression Inventory-II (BDI-II): Preliminary psychometric data with two nonclinical samples. European Journal of Psychological Assessment. 2011;27(4):258-64.

70. Mahoney FI, Barthel DW. Funcional evaluation: the Barthel Index. Maryland state medical journal. 1965;14:61-5.

71. Sequeira C. Cuidar de idosos dependentes. Coimbra, Portugal: Quarteto Editora; 2007.

72. Lawton MP, Brody EM. Assessment of older people: self-maintaining and instrumental activities of daily living. The Gerontologist. 1969;9(3):179-86.

73. Madureira S, Moleiro C, Verdelho A, Guerreiro M. Escala de Atividades Instrumentais de Vida Diária (AIVD). In: Simões MR, Santana I, Grupo de Estudos de Envelhecimento Cerebral e Demência, editors. Escalas e Testes na Demência, 3ª Edição. Lisboa, Portugal: Novartis; 2015. p. 140-5.

For peer review only

For peer review only - http://bmjopen.bmj.com/site/about/guidelines.xhtml

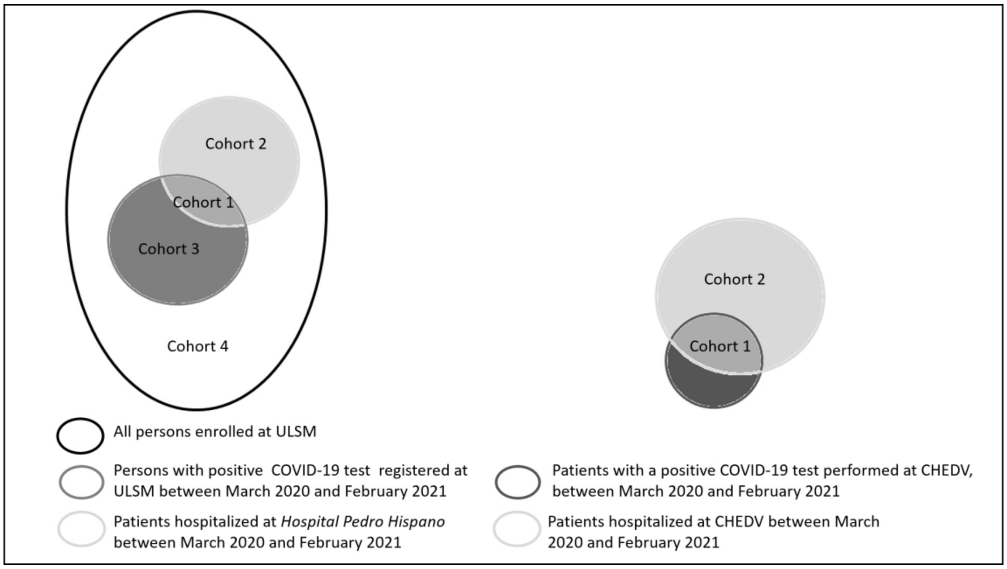

Figure 1. Identification of potential participants from the lists of COVID-19 test results and hospitalization episodes during the period from March 2020 to February 2021, and from the list of individuals enrolled at primary healthcare units of Unidade Local de Saúde de Matosinhos (ULSM). Cohort 1 includes individuals with a positive diagnosis test for COVID-19 registered at ULSM and hospitalized at Hospital Pedro Hispano and those with a positive test performed at CHEDV and hospitalized at one of the three hospitals of CHEDV. Cohort 2 includes individuals with no positive diagnosis test for COVID-19 and hospitalized at Hospital Pedro Hispano and at CHEDV. Cohort 3 includes individuals with a positive diagnosis test for COVID-19 registered at ULSM and with clinical follow-up in the community. Cohort 4 includes individuals enrolled at ULSM with no positive diagnosis test for COVID-19 and without any hospitalization episode between March 2020 and February 2021.

Note: Areas are not proportional to the number of cases.

337x189mm (144 x 144 DPI)
